# Bioinformatics identification of new targets for improving low temperature stress tolerance in spring and winter wheat

**DOI:** 10.1186/s12859-017-1596-x

**Published:** 2017-03-16

**Authors:** Alain B. Tchagang, François Fauteux, Dan Tulpan, Youlian Pan

**Affiliations:** 1 Information and Communications Technologies, National Research Council Canada, Ottawa, ON K1A 0R6 Canada; 2 Information and Communications Technologies, National Research Council Canada, Moncton, NB E1A 7R1 Canada

**Keywords:** Wheat, Breeding, Cold acclimation, Cold stress, Cold tolerance, Low temperature, Marker, Target, Transcriptional regulatory network

## Abstract

**Background:**

Phenotypic studies in Triticeae have shown that low temperature-induced protective mechanisms are developmentally regulated and involve dynamic acclimation processes. Understanding these mechanisms is important for breeding cold-resistant wheat cultivars. In this study, we combined three computational techniques for the analysis of gene expression data from spring and winter wheat cultivars subjected to low temperature treatments. Our main objective was to construct a comprehensive network of cold response transcriptional events in wheat, and to identify novel cold tolerance candidate genes in wheat.

**Results:**

We assigned novel cold stress-related roles to 35 wheat genes, uncovered novel transcription (TF)-gene interactions, and identified 127 genes representing known and novel candidate targets associated with cold tolerance in wheat. Our results also show that delays in terms of activation or repression of the same genes across wheat cultivars play key roles in phenotypic differences among winter and spring wheat cultivars, and adaptation to low temperature stress, cold shock and cold acclimation.

**Conclusions:**

Using three computational approaches, we identified novel putative cold-response genes and TF-gene interactions. These results provide new insights into the complex mechanisms regulating the expression of cold-responsive genes in wheat.

**Electronic supplementary material:**

The online version of this article (doi:10.1186/s12859-017-1596-x) contains supplementary material, which is available to authorized users.

## Background

Low temperature (LT) stress affects the productivity of cereal and other crop plants, most importantly in temperate regions [1]. Plants react differently to chilling (0–15 °C) and freezing (<0 °C) temperatures. Cold acclimation and the acquisition of freezing tolerance are achieved through exposure to chilling, non-freezing temperatures [1–4]. This process increases freezing tolerance and it is associated with complex biochemical and physiological changes [1–5]. These changes are mediated by differential expression of multiple genes [6–9]. Experimental studies suggest that such genes are induced either by cold, or by the state of relative dehydration that is a consequence of cold stress [10]. Several known cold-regulated genes have been identified in gene expression studies. In Arabidopsis, for example, hundreds of genes are differentially expressed in response to cold [11–14]. In temperate grasses including perennial ryegrass [15], barley [16], and wheat [7, 17–20], the expression of many genes is also altered in response to cold.

Specific functions have been assigned to a number of cold-regulated genes, for example transcription factors (TFs) that act as regulators in cold acclimation, or effector molecules that mitigate the potential damage caused by cold stress. However, the specific molecular function of many cold-responsive genes has not yet been investigated, and their role in cold acclimation is unknown [21]. Deciphering the mechanisms of low temperature (LT) response and the specific role of key genes involved in cold stress signaling is crucial for the development of cold-tolerant crops. Gene expression data analysis can suggest genes whose encoding proteins can confer significant influence on the plant phenotype under specified conditions. The identified genes provide the basis for establishing targeted functional markers, which can be used in assisted breeding [22, 23].

To identify genes involved in the wheat response to cold, we examined global changes in the expression of 55,052 unique putative genes represented on the Affymetrix GeneChip Wheat Genome Array (http://www.affymetrix.com/). A total of 90 samples from two winter cultivars (Winter Manitou and Winter Norstar) and two spring cultivars (Spring Manitou and Spring Norstar) [3] were re-analyzed using three different computational methods. Candidate genes were further validated in a distinct dataset comprising two winter cultivars (Harnesk and Solstice) and one spring cultivar (Paragon) [24].

## Methods

**Fig. 1:**
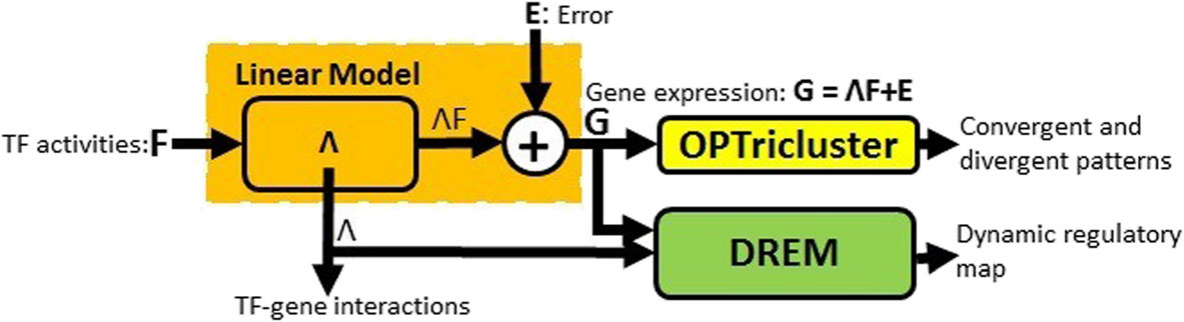
Illustration of the relation between the methods used in this study. The output of the linear model is fed into the inputs of the OPTricluster and the DREM algorithms

The goal of this exploratory study was to identify cold responsive genes in different wheat cultivars using network-based approaches (focusing on cold-responsive TFs and their target genes (TG)). Our approach can be divided into three distinct components. *i*) inference of TF-TG interactions based on a linear model [25], *ii*) identification of bifurcation points in time series and identification of TFs regulating these transitions [26], and *iii*) identification of temporal patterns using a 3D clustering approach [27] (Fig. 1).

### Gene expression data

Microarray data were downloaded from the Gene Expression Omnibus (GEO) database (www.ncbi.nlm.nih.gov/geo/). Experiments were performed using the Affymetrix GeneChip Wheat Genome Array (http://www.affymetrix.com/). The dataset GSE23889 is a time series experiment of 4 wheat genotypes (Winter Manitou (wM), Winter Norstar (wN), Spring Manitou (sM) and Spring Norstar (sN)). The crown of wheat plants were sampled at 8 time points (0, 2, 14, 21, 35, 42, 56 and 70 days), each time point having 3 biological replicates in a random block design, corresponding to a total of 96 samples. The dataset GSE11774 corresponds to a time series experiment of 3 wheat genotypes (Winter Harnesk (wH), Spring Paragon (sP) and Winter Solstice (wS)). The crown and leaves were sampled at 3 time points (3, 5 and 9 weeks post germination), each time point having 3 biological replicates, for a total of 42 samples. Detailed information on experimental protocols and procedures relative to the datasets are available in [3] and [24], respectively.

Wheat TFs were downloaded from the Plant Transcription Factor Database (PlantTFDB) [28]. The current version (3.0) contains 1940 wheat TFs classified into 56 families. Additional TFs and their respective TGs were compiled from the literature [1–21, 24]. Known TF-TG associations were catalogued into a matrix Λ_L_, and used as prior knowledge in our subsequent analysis. Only the interactions that were identified experimentally with *p*-values < 1e-03 were retained. The entries in Λ_L_ were discretized to either ±1 or 0, for representing positive, negative and null TF-gene associations. Wheat gene annotations and ontologies were obtained from Mapman [29], Ensembl Plants [30], Gene Ontology (GO) [31] and the Plant Expression Database (PLEXdb) [32].

### Linear model

Regulatory TF-TG associations were modeled using the linear relationship described in Eq. 1. This linear model was previously used to model transcriptional regulation in *S. cerevisiae* [25] and in *Arabidopsis thaliana* [33].

1 $$ A=\varLambda F+ E, $$

A (*n* × *m*) is the gene expression matrix; Λ (*n* × *k*) is the connectivity matrix; F (*k* × *m*) is the TF activities (TFA) matrix; E (*n* × *m*) is the error matrix; *n* is the number of genes, *m* the number of experimental time points, and *k* the number of TFs. In Eq. 1, Λ, F and E are unknown, and thus have to be inferred from the gene expression matrix A. Similar approaches have been used in [25, 33, 34]. It is considered a realistic approximation of transcriptional regulatory mechanisms when the system reaches a quasi-steady state in the logarithmic space. The quasi-steady-state approximation (QSSA) in chemical kinetics is a mathematical way of simplifying the differential equations that describe chemical kinetic systems [35].

Several algorithms have been developed to address this problem [25, 33, 34, 36, 37]. Here we used a modified version of the reverse engineering algorithm recently described in [33]. This algorithm has three main steps. In the first step, the initial connectivity matrix Λ is constructed, and values are used to initialize the TFA matrix F. In the second step, an alternating least square algorithm [37] is used to update the values of Λ and F sequentially until the convergence of F (*i.e.* the sum of squared changes of F is smaller than a cutoff value *ε* or ||F^*i*-1^ – F^*i*^|| < *ε*, with F^*i*-1^ and F^*i*^ the values of F at (*i*-1)^th^ and *i* ^th^ iterations, respectively). Finally the relationship between the TFs and putative TGs are fine-tuned using a variable selection algorithm [25, 34, 36, 37]. Only TF-TG associations for which the *p-*value is lower than a user defined threshold (p_0_) are retained. The elastic-net algorithm [36] is used to obtain a sparse connectivity matrix, which is characteristic of many biological systems [34]. Ridge regression is used to overcome numerical instability during the updating step [38]. The main difference between this algorithm and that in [33] is in the initialization of the connectivity matrix Λ. Here, the initial values of Λ are a combination of the inferred values from literature and from gene expression data (expression level of the TFs), whereas in [33] values were inferred from the literature only. Integration of the expression level of TFs in the initialization step is shown to improve the TF-TG relationships, compared to other versions of the algorithms [25, 33, 37], which did not account for the expression level of TFs. Similar improvements were also observed within the different version of the Dynamic Regulatory Map algorithms [26].

### Dynamic regulatory maps

The Dynamic Regulatory Event Miner (DREM) [26] was used to reconstruct regulatory events happening in wheat under cold stress. DREM takes time series gene expression data and TF-TG interactions as input and produces an annotated dynamic regulatory map that highlights bifurcation events where the expression of a subset of genes diverges from the rest of genes. Each bifurcation event is associated with a set of TFs that selectively regulate these events.

### OPTricluster

The Order Preserving Triclustering Algorithm (OPTricluster) [27] was used to model the three dimensional features in the dataset. For example, in the dataset GSE23889, the three dimensions are G × S × T, where **G** = **{g**_**1**_, **g**_**2**_, …, **g**_**n**_ **}** is the set of *n* probe sets on the microarray, **S = {wM**, **wN**, **sM**, **sN}** the set of *l* (4) wheat genotypes and T = {0, 2, 14, 21, 35, 42, 56, 70} the *m* (8) time points. The three dimensional data was used as input for the OPTricluster algorithm, which outputs a set of 3D conserved and divergent patterns and associated *p*-values. Each conserved pattern corresponds to a group of genes with similar behavior across subsets of time points and in subsets of genotypes. These conserved signals correspond to potential genetic pathways with similar expression patterns within a certain time frame and in subsets of wheat cultivars. On the other hand, divergent patterns correspond to groups of genes that behave differently in subsets of genotypes, thus representing potential markers that differentiate genotypes.

### Gene ontology and pathway analysis

The Plant Orthology Browser [39] was used to identify orthologs in other plant species, GOAL [40] was used for Gene Ontology (GO) enrichment analysis and REACTOME [41] was used for pathways analysis.

## Results

Results were obtained by analyzing the dataset GSE23889 [3], which contains four wheat cultivars: **wM**, **sM**, **wN** and **sN**. These results were validated using the dataset GSE11774 [24], which contains three wheat cultivars: **wH**, **sP** and **wS**.

Gene expression data was normalized using the Robust Multi-Array normalization algorithm (RMA) and log_2_ transformed. Prior to analysis, genes whose expression level did not change by at least 2 fold in any time interval based on the difference between the maximum and minimum levels of gene expression from 0 to 70 days of cold treatment were filtered out. As described by Laudencia-Chingcuanco et al. [3], a probeset was deemed to represent a unique wheat gene and the signal intensities in that probeset to represent the expression of the corresponding gene. Recent probeset annotations were obtained from the wheat oligoarray database at http://www.plexdb.org.

### Transcriptional regulatory network underlying low temperature stress

For this analysis, we used the combined gene expression data A (N × M) of the 4 wheat cultivars **wM**, **sM**, **wN** and **sN**. The initial TF-TG interactions information Λ(N × K) = [Λ_L_ Λ_T_] was curated based on the literature and gene expression data as we described earlier. *N* = 61,290 is the number of probes on the wheat Affymetrix 61 K chip (representing 55,052 potential unique genes in the wheat genome), M = 32, the number of combined experimental time points of the four cultivars (each has 8 time points), and K = 109, the number of TFs, selected from the initial list of 1940 wheat TFs, whose interactions with wheat genes under LT, cold or freezing stress were found through either literature exploration or computational approaches. Literature exploration was based on the fact that the TF had been documented with experimental evidence as regulator of cold responsive genes. Computational approaches involved differential and co-expression across subsets of the time points of a TF and its regulatory association with potential TG, and annotations to similar or related biological processes.

The linear model identified 47 TFs (Table 1) involved in the wheat response to cold stress, which interact with a total of 2789 genes, with *p*-values < 1e-03. The 47 cold-responsive TFs are grouped into 12 families (Table 1). The linear model showed that AP2/ERF, bHLH, MYB, C2H2 zinc finger, RR, PRR and WRKY families play the most significant roles during cold stress response in wheat, as it has been shown in other plant species such as Arabidopsis and rice [12–14].

**Table 1:** List of TFs inferred by the linear model to play a regulatory role during cold stress with significant *p*-values (<1e-03)

TF family	TFs	Symbol	Description	References	*p*-value
AP2/EREBP, APETALA2/Ethylene-responsive element binding protein family	Ta.27144.1.S1_A_at	RAP2.12	RAP2.12, RELATED TO AP2 12	New, [60]	5.0e-09
	TaAffx.122374.1.A1_at	CBFIVa-A2	Cluster: CBFIVa-A2; *n* = 1; Triticum aestivum	[6, 24]	1.0e-12
	TaAFFX.98930.1.A1_at	CBFIIId-12	DREB1A, CBF3, ATCBF3 | dehydration response element B1A	[6, 10, 24]	1.0e-12
	Ta.22822.1.S1_X_at	RAP2.3	RAP2.3, ATEBP, ERF72, EBP | ethylene-responsive element	New, [60]	1.0e-12
	TaAFFX.1024.1.A1_at	CBFIIId-B19	DREB1A, CBF3, ATCBF3 | dehydration response element B1A	[6, 10, 24]	1.0e-12
	Ta.2781.1.S1_at	CBF1	AP2/EREBP type transcription factor; *n* = 1	[6, 10, 24]	1.0e-12
	Ta.25843.1.A1_at	CBF2	C repeat-binding factor 2	[6, 10, 14, 24]	1.0e-12
	Ta.13471.1.S1_at	CBFII-5.2	Dehy-responsive element-binding protein 1C	[6, 10, 24]	5.0e-08
	TaAffx.15675.1.A1_at	TmCBF7	TmCBF7; *n* = 1; Triticum monococcum	[24]	5.0e-08
	TaAffx.114399.1.S1_s_at	CBFIIIc-B10	CRT/DRE binding factor 10	[6, 10, 24]	5.0e-08
	TaAffx.86939.1.S1_at	HvCBF7	HvCBF7; *n* = 1; TINY2; encodes a member of the DREB subfamily	[24]	1.0e-03
	Ta.350.1.A1_s_at	TaCBF6	Cluster: TaCBF6; *n* = 1; Triticum aestivum	[24]	5.0e-08
	TaAffx.43752.1.A1_at	CBFIIIc-D3	Cluster: CBFIIIc-D3; *n* = 1; Triticum aestivum	[6, 10, 24]	5.0e-08
	TaAffx.28024.1.S1_at	CBFIVd-B9	Dehy-responsive element-binding protein 1B	[6, 24] no	1.0e-03
	Ta.5304.1.S1_at	RAP2.1	AP2 domain containing protein RAP2.1	[6]	1.0e-03
	TaAffx.23008.1.S1_at	RAP2.6	related to AP2 6 l	[6]	1.0e-03
bHLH,Basic Helix-Loop-Helix family	TaAFFX.18844.1.S1_at	ICE1	inducer of CBF expression 1	[6, 24, 51]	1.0e-12
	TaAffx.12752.1.S1_s_at	ICE2	SCRM2, ICE2 | basic helix-loop-helix (bHLH)	[6, 51]	6.3e-08
	TaAffx.12752.1.S1_at	ICE2	inducer of CBF expression 2	[6, 51]	6.3e-08
HB,Homeobox TF	Ta.6874.1.A1_at	HOS3	HOS3 protein	[66]	1.6e-10
CCAAT box binding factor	TA.27414.1.S1_AT	NF-YA6	NF-YA6 | nuclear factor Y, subunit A6	New	1.0e-03
	TA.1817.1.S1_AT	NF-YA5	NFYA5, NF-YA5 | nuclear factor Y, subunit A5	New	1.0e-03
MYB domain transcription factor family	TA.5251.1.A1_AT	MYBAS1	Myb-related protein MYBAS1	New	1.0e-03
	TA.26049.1.S1_A_AT	MYB1/MYB4	Transcription factor Myb1	[55]	1.0e-03
	TA.5405.1.S1_X_AT	MYB15	MYB15 | myb domain protein 15	[6, 24]	3.9e-04
MYB-related transcription factor family	TA.25744.1.S1_AT	EPR1	EPR1, RVE7 | Homeodomain-like superfamily	New	7.9e-07
	TA.7524.1.A1_AT	LHY	LHY | Homeodomain-like superfamily protein	New	1.0e-03
	TA.8661.1.S1_AT	MCB2	MCB2 protein	New	1.0e-03
WRKY domain transcription factor family	TA.16082.1.A1_A_AT	WRKY1	WRKY transcription factor; *n* = 1	New, [58, 59]	
	TA.16082.1.A1_AT				1.0e-03
	TA.4678.2.S1_AT	WRKY1	WRKY transcription factor; *n* = 1	New, [58, 59]	1.0e-03
	TAAFFX.64818.1.S1_AT	WRKY48	WRKY48 | WRKY DNA-binding protein 48	New, [58, 59]	1.0e-03
Pseudo Regulatory Response (PRR )	TA.13464.1.S1_S_AT	PRR1	Two-component response regulator-like PRR1	[56, 57]	1.0e-03
	TA.29499.1.A1_AT	PRR1	Two-component response regulator-like PRR1	[56, 57]	1.0e-03
Regulatory response (RR)	Ta.10408.1.S1_at	RR6	Cluster response regulator 6	[52]	7.9e-07
	Ta.11777.1.S1_X_at	RR2	Cluster: ZmRR2 protein	[52]	7.9e-07
Chromatin Remodeling	TA.16121.2.S1_AT	SWI/SNF	SWI/SNF-related matrix-associated actin-dependent regulator of chromatin	New	1.5e-11
AS2	Ta.7384.2.S1_A_at	DUF260	Seed specific protein Bn15D17A, DUF260	[6]	1.0e-03
	TaAFFX.3993.1.S1_at	DUF260	Cluster: Predicted protein; *n* = 1, DUF260	[6]	1.0e-03
C2H2 zinc finger family	TA.103.1.S1_AT	ZAT10	Cluster: Zinc finger protein 1; *n* = 1	[6]	6.3e-08
	TA.29449.1.S1_S_AT	ZOS3	Cluster: Zinc finger protein 1; *n* = 1	[6, 24]	6.3e-08
	TaAffx.130052.1.S1_at	ZAT12	Cluster: C2H2 zinc finger protein; *n* = 1	[6, 14]	1.0e-03
	TAAFFX.98004.1.S1_AT	A20/AN1-like	A20/AN1-like zinc finger family proteinSTRESS ASSOCIATED P1	[66]	1.0e-03
	Ta.6076.1.S1_at	ESK1	ESK1, TBL29 | Plant protein of unknown function (DUF828)	[10, 54]	1.0e-12
	Ta.7191.1.A1_at	ELO2	ELO2, ABO1 | IKI3 family protein	[66]	1.6e-10
	Ta.26723.1.A1_at	SIZ1	ATSIZ1/SIZ1, putative, expressed	[6]	1.0e-03
	Ta.13279.1.S1_a_at	EIN3	EIN3-binding F box protein 1	[52]	1.0e-03
	TaAffx.36731.1.S1_at	EIN4	Protein EIN4 *n* = 1 Tax = Arabidopsis thaliana	[52]	1.0e-03
	TaAffx.32271.2.S1_at	LOS2	Enolase; *n* = 1; Oryza sativa Indica Group	[6]	1.0e-03
	Ta.26244.1.S1_at	MSL5	mechanosensitive channel of small conductance-like 5	New	7.9e-07
	Ta.7091.1.S1_at	P5CS2	P5CS2 | delta 1-pyrroline-5-carboxylate synthase 2	[10, 49]	1.0e-03

“New” entry in column References indicates that the association between this TF and cold stress is a new discovery in this study

**Fig. 2:**
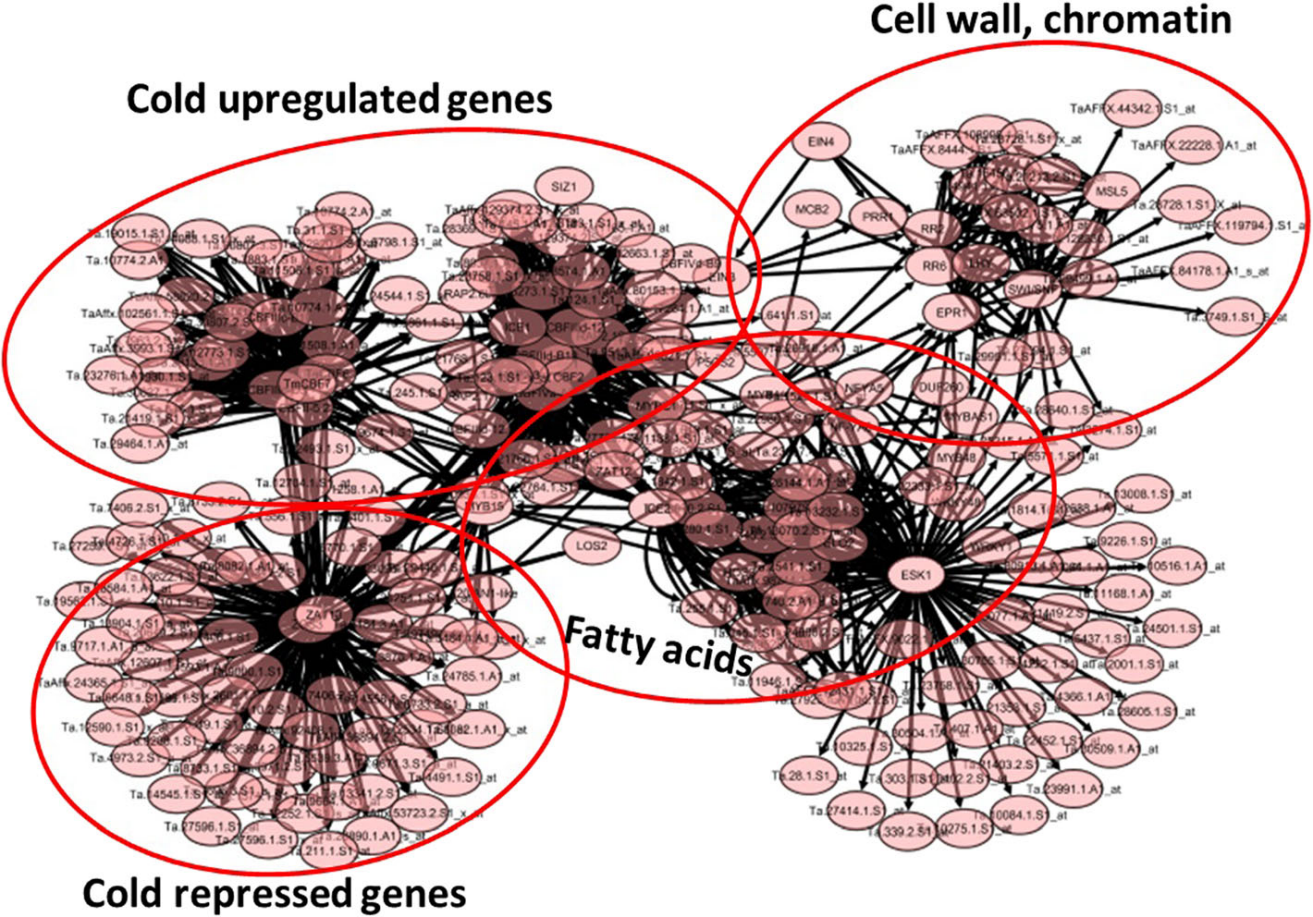
Core TF-genes interaction model similar to the seven wheat cultivars under low temperature stress, showing how cold stress, lipids and FA, cell wall and chromatin and cold downregulated genes are respectively clustered together and the relationships between them. Detailed individual associations shown in this figure are available in the Additional file 1. This network was obtained using Additional file 1 and Cytoscape (http://www.cytoscape.org/)

**Fig. 3:**
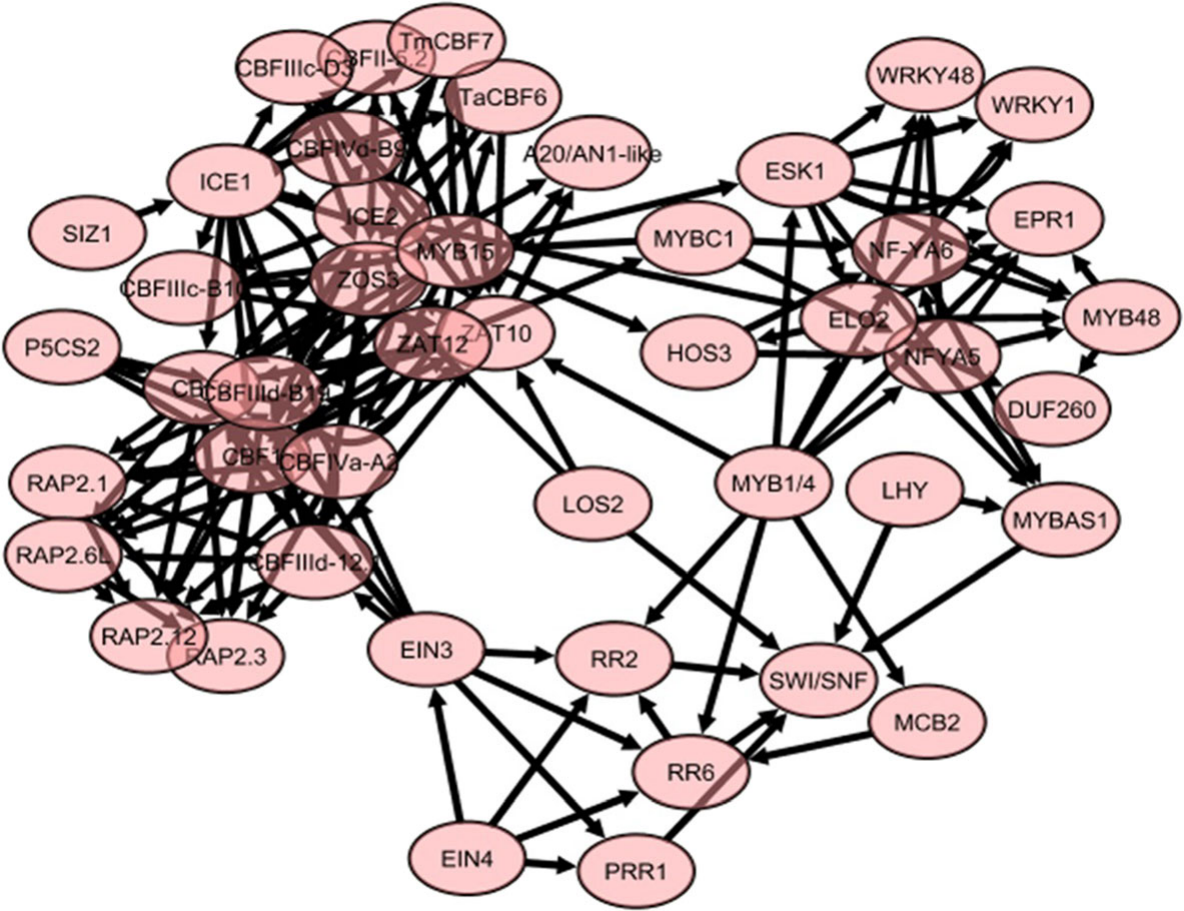
Core TF-TF interactions model similar to the seven wheat cultivars under low temperature stress. It shows a high connectivity between the CBFs. This network was obtained using only the TF-TF associations in the Additional file 1 and Cytoscape (http://www.cytoscape.org/)

**Fig. 4:**
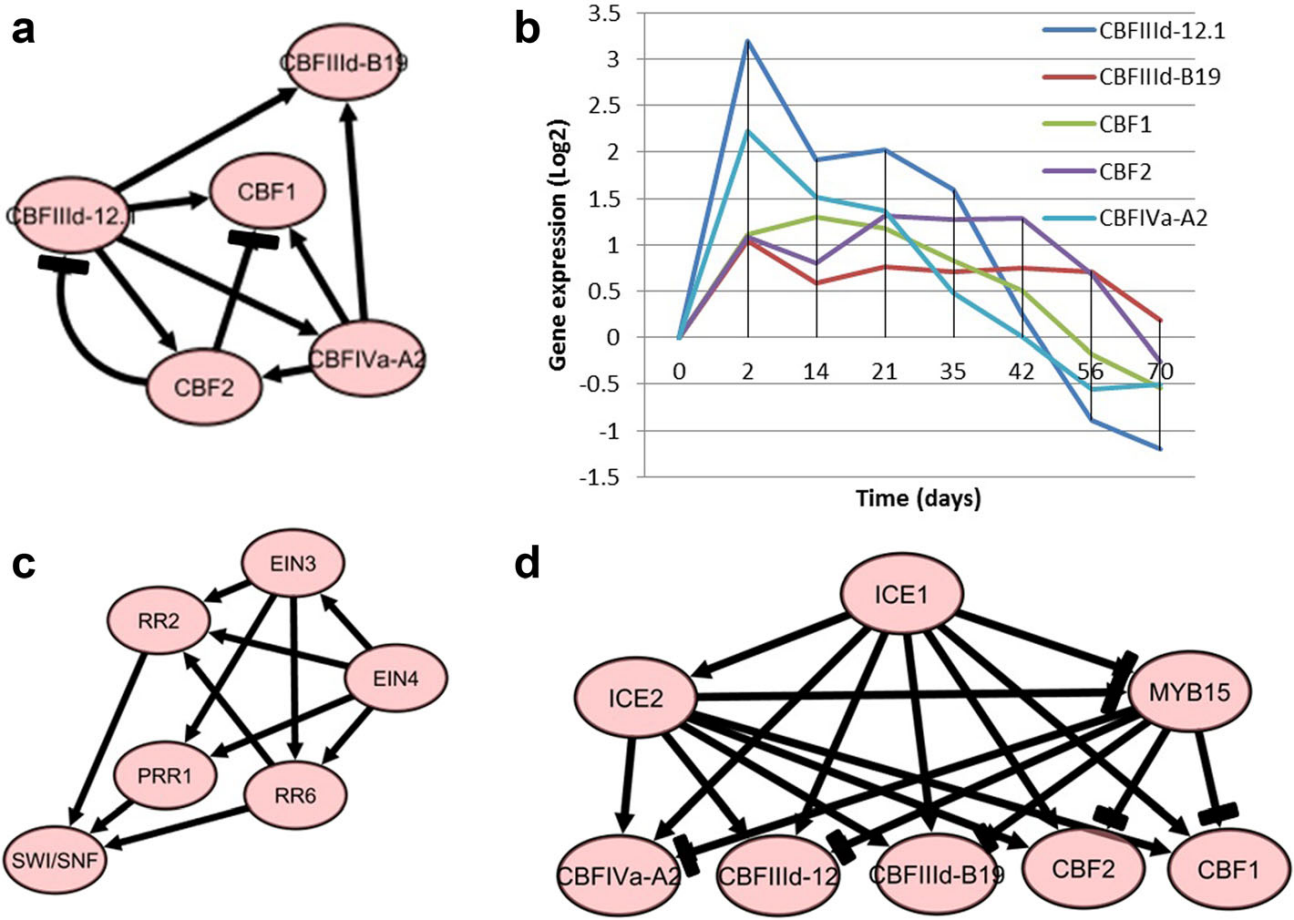
Example of small world network inferred from the core TF-TF network. **a** High connection among five CBFs. **b** Expression/regulatory activity of five CBFs in (**a**). **c **High connections among EIN3, EIN4, RR2, RR6, PRR1 and SWI/SNF. **d** High connections ﻿among﻿ ICE1/2, MYB15 and CBFs. This subnetwork was obtained using only the corresponding TF-TF associations in the Additional file 1 and Cytoscape (http://www.cytoscape.org/)

Figures 2 and 3 show the inferred TF-gene and TF-TF networks common in the four wheat cultivars respectively. Detailed statistical and biological analysis of the network for each wheat cultivar revealed significant patterns and regulatory information. We found nearly 2.5% of the inferred connectivity matrix to be non-zero. Only eleven TFs (ICE1, ICE2, CBFIIId_12.1, CBFIIId-B19, CBF1, CBF2, CBFIVa-A2, MYB15, MYB4, ESK1 and ELO2) were shown to interact with more than 50 genes. Other parameters that can be used to describe a biological network include the clustering coefficient and the scale-free topology criterion [42]. The clustering coefficient measures the “small-world” property in the network, which in this study is the likelihood that two connected TFs are also connected to a common TF. Examples extracted from the TF-TF network (Fig. 3) are shown in Fig. 4. In the sub-network of Fig. 4a, CBFIIId_12.1, CBFIIId-B19, CBF1, CBF2, and CBFIVa-A2 interact with each other (with a clustering coefficient of 0.867) to control the expression of cold-regulated genes (Fig. 2). Furthermore, in this sub-network it is shown that CBFIIId_12.1 and CBFIVa-A2 act upstream of CBFIIId-B19, CBF1 and CBF2, respectively (Fig. 4b), and that CBF2 acts as a negative regulator of CBF1 and CBF3. Similar observation has been experimentally shown in Arabidopsis [11]. In the sub-network of Fig. 4c (with a clustering coefficient of 0.6), EIN4 interacts with EIN3 to control the regulation of response regulator (RR) and pseudo-response regulator (PRR) TFs. In the sub-network of Fig. 4d, ICE1, ICE2, and MYB15 interact with each other with a clustering coefficient of 0.82 to control CBFs in Fig. 4a. The TF-gene interactions list can be found in the Additional file 1.

### Transcriptional regulatory events following low temperature stress

**Fig. 5:**
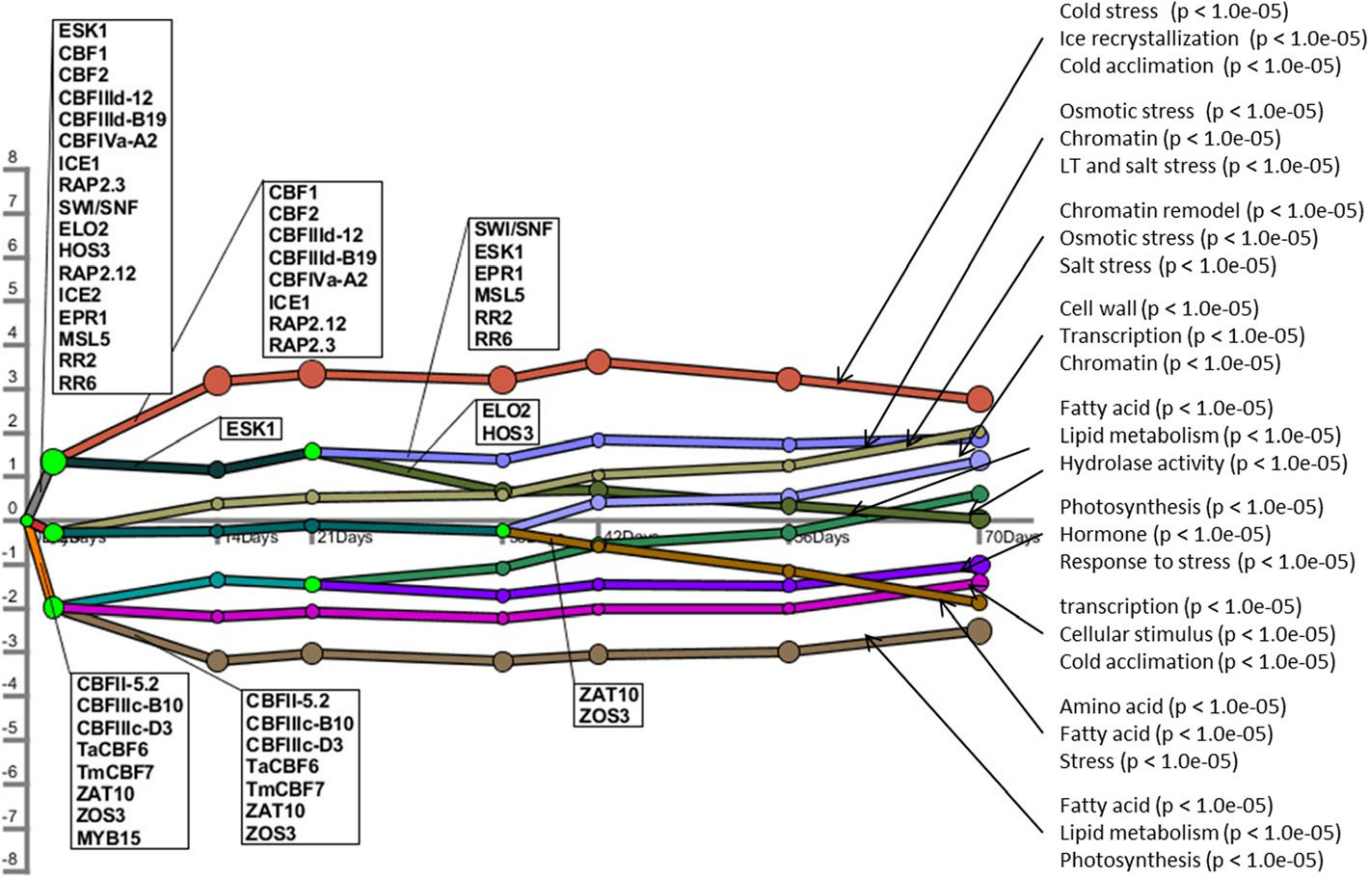
Dynamic regulatory map of Winter Manitou. The x-axis is the time-points in days. The y-axis is the gene expression levels. This map contains 10 paths. Each path corresponds to the mean of the fold change of expression level of the genes that belong to it, relative to day 0. The size of nodes corresponds to standard deviations of the fold changes. This map is obtained using the TF-gene interactions (Fig. 2, Additional file 1) and the gene expression data of the Winter Manitou as input to the DREM algorithm, using a TF split cutoff score of 0.005

The TF-TG interaction data (Fig. 2 and Additional file 1) was used to reconstruct a set of regulatory events during cold stress using a procedure for learning input-output hidden Markov models. Figure 5 presents the temporal map of wM. Additional file 2: Figures S1, S2 and S3 present the temporal maps of **wN**, **sM** and **sN** respectively. The maps of **wM**, **wN**, **sM**, and **sN** contained 10, 10, 14, and 13 unique paths, respectively, controlled by a total of 25 TFs (using a TF split cutoff score of 0.005). In some cases, the same TF (e.g. CBF1, CBF2 and ESKI) appears multiple times on the same path and is significantly associated with multiple bifurcations, indicating a continuous regulatory function in the entire time series.

In all the cultivars, following cold stress, there was a massive transcriptional response involving either activation or repression. The Dynamic Regulatory Event Miner (DREM) algorithm assigned several TFs to the first bifurcation. Seventeen TFs (ESK1, CBF1, CBF2, CBFIIId-12, CBFIII-B19, CBFIVa-A2, ICE1, RAP2.12, RAP2.3, SWI/SNF, ELO2, HOS3, ICE2, RR6, RR2, EPR1, and MLS5) were associated with genes up-regulated upon cold stress. As shown in Fig. 5 and Additional file 2: Figures S1, S2 and S3, GO analysis of these genes (up-regulated paths) showed that they were highly enriched for terms related to cold stress with *p*-values < 1e-05. We observed a major increase in the activity of cold-regulated genes (WCOR14a, WCOR14c, WCOR1418, WCOR80, CS120, CS66, CS6, CR14, WCOR518, WCOR615 and WCOR413), the fatty acid biosynthesis genes (FAR1, FAR4 and FAR5), late embryogenesis abundant (LEA) genes, and those linked to *proline* and *dehydrin* activities.

Eight TFs (ZAT10, ZOS3, CBFII-5.2, CBFIIIc-B10, CBFc-D3, TaCBF6, tmCBF7 and MYB15) associated with the down-regulated paths (Fig. 5 and Additional file 2: Figures S1, S2 and S3) were conversely associated with cold-repressed genes. GO analysis of these repressed genes showed that they were associated with terms related to fatty acid, lipid metabolism and cold stress with *p*-value < 1.0e-04. In fact, we observed a major decrease in the expression of genes linked to fatty acids (FAD7, FAAH, DGK5 and SQD2), *jasmonate*, *jacalin*, *photosynthesis*, *defensin*, and *dirigent* (DIR). Two TFs (ZAT10 and ZOS3) were associated with groups of genes that were neither up nor down-regulated at the onset of cold exposure, but were shown to be differentially expressed 35 days later and associated with the regulation of some stress responsive genes, such as Ta.21350.2.S1_at (BBTI11), Ta.9730.1.S1_at (heat stable protein: HS1), and lipid genes such as Ta.8082.1.A1_at (Cyclopropane-fatty-acyl-phospholipid synthase, putative, expressed).

### Similarities and differences between wheat cultivars

**Fig. 6:**
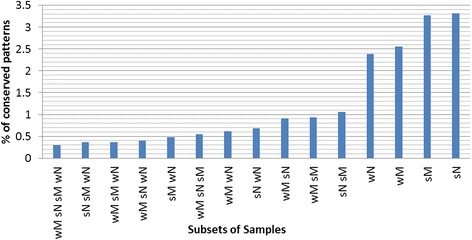
Percentage of genes with similar behavior in two or more cultivars. The x-axis represents the genotype combination, and the y-axis the percentage. This graph was obtained by plotting the statistics of the conserved and divergent patterns yielded by the OPTricluster algorithm

Analysis of the linear model indicated that the number of TGs of each TF varies significantly across cultivars. Similarly, Fig. 5 and Additional file 2: Figures S1, S2 and S3 show that the transcriptional regulatory maps of the four cultivars (**wM**, **wN**, **sM**, and **sN**) are different. To study the similarities and differences across the four cultivars in term of gene expression, we used the OPTricluster algorithm as described in the Methods section and identified differential expression of 1570 (2.56%), 2002 (3.27%), 2027 (3.31%), and 1465 (2.39%) genes in **wM**, **sM**, **sN**, and **wN**, respectively, with a 2-fold change across the eight experimental time points (Fig. 6). Among these genes, only 182 (0.3%) changed similarly across the four cultivars, whereas, 163, 322, 440 and 422 were uniquely differentially expressed in **wM**, **wN**, **sM** and **sN**, respectively.

#### Conserved patterns: potential genetic pathways

Conserved patterns represent groups of genes that are co-expressed among the cultivars and across the experimental time points and may be co-regulated by the same group of TFs. Additional file 2: Table S1 shows a group of 100 genes that were identified by the OPTricluster algorithm to be highly conserved (*p*-value < 1e-04) and up-regulated across the cultivars. GO analysis of these genes showed that they are enriched for terms such as cold acclimation, cold-regulated protein, cold shock, ice recrystallization and LT stress, with *p*-values < 1e-05. Additional file 2: Table S2 shows another group of genes that were also highly conserved (*p*-value < 1e-04) and down-regulated in the four cultivars. GO analysis of this group revealed relevant terms such as *jacalin*-like, *dirigent*, *defensin*, and cold-regulated with *p*-values < 1e-04.

Several genes related to fatty acid (FA) metabolism also exhibited a conserved behavior in the four cultivars. Additional file 2: Figure S4 presents a cluster of 20 genes related to various aspects of FA and lipid metabolism. These genes were differentially expressed at the onset of cold stress and behaved similarly across the time series. Among these genes, fatty acyl CoA reductases FAR1, FAR4 and FAR5 are involved in the synthesis of very long chain fatty acids (VLCFAs), which play important roles in cold stress [43]. Other important genes in cold acclimation included Ta.13232.1.S1_at (C-4 sterol methyl oxidase, putative) and TaAffx.107979.1.S1_at (Sterol 14-demethylase), which belongs to the Sterol desaturase family. Conversely, there was also a significant decrease in the expression level of 12 genes related to various aspects of FA and lipid metabolism (Additional file 2: Figure S5). GO analysis of these genes showed that they are involved in the desaturation of linoleic acid (18:2; 18) to alpha-linolenic acid (18:3; 18), which is another important process in plants during cold acclimation [44].

#### Divergent patterns: potential markers or targets

In terms of differences between the four cultivars, it is interesting to see how the two spring cultivars had higher number of differentially expressed genes as compared with the two winter cultivars. Among divergent genes, 163, 322, 440, and 422 were uniquely expressed in **wM**, **sM**, **sN**, and **wN** respectively, with the **sN** having the highest number and the **wN** the lowest. A list of 127 divergent genes (Additional file 2: Table S3) were selected and ranked based on their co-expression among the different cultivars and their membership in the cluster corresponding to cold stress and GO enrichment analysis. They represent potential cultivar-specific cold stress markers.

Collectively, three types of divergent patterns were inferred by the OPTricluster algorithm. The first type of divergent patterns is shown in Additional file 2: Figure S6. A group of genes has an expression pattern unique to the **wN**, whereas levels increase at 56 days by more than 4 fold compared to **wM**, **sM** and **sN**. This group of genes was also confirmed by the DREM algorithm (Additional file 2: Figure S1). GO and orthology analyses showed that a majority of these genes had no assigned function or ortholog in other species. Only 4% of the genes (3 out of 73) in these divergent clusters had a known function, namely TaAffx.124056.1.S1_at (photosynthesis light reaction/cyclic electron flow-chlororespiration), TaAffx.6593.1.A1_at (protein/synthesis/ribosomal protein/prokaryotic/chloroplast/50S subunit/L33) and Ta.10297.2.S1_at (cell wall/pectin esterases/PME). The linear model did not identify any TFs associated with these genes.

A second type of divergent patterns is shown in Additional file 2: Figure S7. Two genes display a high expression level at the onset of cold stress in **wN** compared to **wM**, **sM** and **sN**: the *dehydrin* 5/cold-shock protein gene TaAffx.137429.1.S1_at and the cold-regulated gene Ta.25539.1.S1_at. Overexpression of *dehydrin* in plants is known to enhance cold tolerance [45]. Given that **wN** is a winter cultivar and that these two genes are uniquely over-expressed in this cultivar, we consider them putative markers for cold acclimation unique in **wN**. The third type of divergent patterns is represented by four putative cold-acclimation marker genes in the MADS-box family (Additional file 2: Figure S8). Dissimilarity between the four cultivars is based on the delay in terms of activation or repression of certain group of genes. Genes in this group had previously been shown to be potential makers for breeding [46, 47].

### Classification of unannotated genes

Conserved patterns represent groups of genes that are co-expressed and/or probably co-regulated by the same group of TFs. We associated unannotated genes with co-expressed and putatively co-regulated genes, which are known to have specific GO terms, pathways and regulation by TFs. Genes with an unknown function that appears to have a similar expression profiles to known genes can be part of the same biological pathways [48]. Additional file 2: Figure S9 shows an example of 6 genes (in 9 probes) that are up-regulated at the onset of cold stress and co-regulated by the same TFs (CBFIIId-12, CBFIIId-B19, CBFIVa-A2, CBF1 and CBF2). In this cluster, Ta.2541.1.S1_s_at (COR615) is a cold-responsive LEA/RAB-related COR protein, Ta.21768.1.S1_at is an ice recrystallization inhibition protein 1 precursor and Ta.7091.1.S1_at is a putative delta-1-pyrroline-5-carboxylate synthetase. TaAffx.38397.1.A1_at may be orthologous to the rice gene Os03g08580 which is an expressed protein, whereas no orthologs were found for Ta.22063.1.S1_s_at and TaAffx.97142.1.S1_at. Overexpression of [delta]-pyrroline-5-carboxylate synthetase increases proline production and proline accumulation, which confers cold tolerance in plants [49]. In this example, the three unannotated genes that are co-expressed with the three annotated ones (COR615, Ta.21768.1.S1_at and Ta.7091.1.S1_at) may also be part of the same cold response pathways, and represent potential candidates for further experimental validation. Using this approach, 35 genes were assigned new putative cold-related functions in wheat Table 2.

**Table 2:** List of 35 new cold related genes identified using the co-expression behavior with known and well characterized genes

Probe ID	Description	wM	sN	sM	wN
Ta.10319.1.A1_s_at	Myb-like	4	3.8	5.2	1.7
Ta.10857.1.A1_at	Cluster: Os10g0566400 protein	2.1	2.8	2	2.9
Ta.13134.1.A1_at	Immediate early protein ICP0	4.6	3.9	5	4.2
Ta.13193.1.S1_at	NADH-ubiquinone oxidoreductase	3.7	3.5	3.5	3.2
Ta.13239.1.S1_at	Pherophorin-C1 protein precursor	4.9	4.8	4.8	5.3
Ta.13595.1.A1_at	BQ169949	3	2.8	2.6	2.9
Ta.1722.1.S1_at	TC434908	4	2.7	4	2.5
Ta.18391.1.S1_at	CA635688	1.9	1.7	1.8	1.7
Ta.18720.1.S1_a_at	Gamma-thionin (Defensin-like protein 1)	6.5	6	5.4	4.7
Ta.19327.1.S1_at	Fe(III) dicitrate ABC transporter	3.6	3	3.8	2.8
Ta.22063.1.S1_s_at	TC421941	3.1	3.3	3.2	3.4
Ta.22766.1.S1_a_at	TC445245	6.9	7.3	8	7.3
Ta.23419.1.S1_x_at	High molecular mass early light-inducible protein	2.6	3.5	2.8	2.8
Ta.24761.2.S1_at	TC376085	1.8	1.2	1.7	1.1
Ta.27719.1.S1_at	5-oxoprolinase; *n* = 1; Sphingomonas wittichii	5.2	5.2	5.1	5
Ta.27719.2.S1_x_at	TC448971	2.3	2.2	2.6	2
Ta.7053.1.S1_at	TC427799	4.1	3.7	4	3.8
Ta.7091.1.S1_at	TC37493 (P5CS2)	2.9	2.6	3	2.6
Ta.7934.3.S1_at	HNH nuclease	4.8	3.3	4.3	3.9
Ta.9600.1.S1_x_at	Low molecular mass early light-inducible protein	2.7	2.7	2.8	2.7
TaAffx.134872.1.S1_at	TC434386 (Ycf1)	1.1	1.3	1	1.1
TaAffx.144000.1.S1_s_at	Humulus lupulus 26S ribosomal RNA gene	5.6	2.3	5.8	1.9
TaAffx.34169.1.S1_at	TC396129 (embryonic protein DC-8)	4.5	3.5	3.4	5
TaAffx.73215.1.S1_at	TC407368	3.1	2.7	3.2	2.2
TaAffx.38397.1.A1_at	Express protein similar to Os03g01841100	2.9	3.3	3.0	3.0
TaAffx.97142.1.S1_at	weakly similar to UniRef100_Q2IMJ3	3.6	3.2	2.7	3.6
Ta.13153.1.S1_s_at	Cluster: Biotin synthesis protein	0.2	2.2	0.2	1.4
Ta.13232.1.S1_at	Cluster: Sterol desaturase family protein	2.4	3.4	1.4	3.4
Ta.13232.2.S1_at	Cluster: Sterol desaturase family protein	2	2.1	0.7	4
Ta.13784.1.S1_at	Cluster: BLT14.1 protein	5.4	5.7	4	6.4
Ta.14903.1.S1_at	Cluster: Chalcone synthase	4.6	2.3	4.1	1.7
Ta.19303.1.S1_at	Cluster: Expressed protein	3.1	4.3	2.8	4.9
Ta.28533.1.S1_at	Cluster: PS II 10 kDa protein	1.9	2.7	1.8	4.7
TaAffx.116865.2.S1_at	LTPL114 - Protease inhibitor/seed storage/LTP	4.8	5.3	3.7	6
TaAffx.124475.1.A1_at	Cluster: Hblt14.2 protein	2.9	4.1	1.4	6.4
TaAffx.144000.1.S1_s_at	Humulus lupulus 26S	5.6	2.3	5.8	1.9
TaAffx.34169.1.S1_at	TC396129	4.5	3.5	2.8	5

Numbers in column 3–6 represent the fold change between the min and the max of the expression level across the experimental time points

### Comparative analysis with previous studies

The first goal of our study was to infer the transcriptional regulatory events that happen in wheat during cold stress. Several cold-regulated genes identified in this study had previously been identified and experimentally validated elsewhere: HMGB1, TaGRP2 and DHN14 [20], WCS120, WCS200, WCS180, WCS66 and WCS40 [19], WCOR719, COR14a, WCOR615, WCS19 and WCOR726 [18].

We compared our list of 2789 cold-responsive genes identified using the linear model, the DREM algorithm and the OPTricluster algorithm with the 2771 list of genes reported in [3] to show genotype and time (*g* × *t*) interaction at *p*-value < 0.001. These differentially expressed genes were identified in [3] using gene-specific ANOVA with the following model: *y*_*ijkr*_ = *μ*_*i*_ + *g*_*ij*_ + *t*_*ik*_ + *(g* × *t)*_*ijk*_ + *ε*, using the GeneSpring package. *y* is the response variable and it represents the log_2_ transformed normalized probeset intensity. μ represents the grand mean level of expression for each gene. Variables *g*, *t* and *g* × *t* represent the genotype effect, the effect of time (duration of cold treatment) and the interaction between genotype and time, respectively, and *ε* represents the stochastic error, which is assumed to be normally distributed. The indices *i*, *j*, *k* and *r* indicate the probeset (gene), genotype, time and biological replicates, respectively.

Surprisingly, only 973 genes were similar in both lists. Our analysis excluded the remaining 1798 genes because their expression did not change by at least two fold during cold treatment in any of the four wheat cultivars from 0 to 70 days. However, the analysis of the 1816 genes present in our list but not identified in [3] showed that these genes not only changed significantly during cold treatment, but were also enriched for GO terms associated with cold stress (*p*-value < 1e-05), FA and lipid metabolism (*p*-value < 1.0e-05). Additional file 2: Table S4 shows a partial list of these genes. This list includes cold-responsive genes that are equivalent or similar to WCOR80, CS120, CS66, WCOR518, CS120 and WCOR615. Among these genes, PTACR7, a gene from hard red winter wheat, which is induced by low temperature but not by ABA or stresses such as salt, dehydration or heat [50], exhibited a fold-change of 4 in log2 scale across all the four cultivars.

We also validated the set of genes uniquely identified by our approach using PLEXdb. PLEXdb Gene Oscilloscope tool allows users to search for experiments where expressions of queried genes fluctuate (oscillate) the most. Interestingly, the Gene Oscilloscope confirmed most of our identified cold-related differentially expressed genes. Additional file 2: Figure S10 shows the Gene Oscilloscope results for Ta.123.1.S1_x_at (Cold acclimation protein WCOR80), Ta.124.1.S1_x_at (Cold-shock protein CS120) and Ta.145.1.A1_x_at (Cold shock protein CS66). These results shows that these three probe sets have coefficient of variations of 20, 16, and 17 respectively, corresponding to row TA42 in Additional file 2: Figure S10. These results further confirm the validity of the sets of genes that were selected for further transcriptomics and regulatory network modeling.

The dataset GSE11774 corresponds to the expression profiles of two winter wheat cultivars (winter Soltice ,**wS** and winter Harnesk ,**wH**) and one spring cultivar (spring Paragon, **sP**), sampled from crowns and leaves collected at 3, 5 and 9 weeks [24]. The authors reported 3113 (up = 1711, down = 1402) genes showing greater than two fold change in at least one cultivar after cold shock. The difference in genes identified as cold-responsive may be attributable to the choice of cultivars and sampling times and the part of plant that was analyzed. OPTricluster analysis of this dataset showed that about 200 genes exhibited similar patterns across the three cultivars. Comparative analysis of the combined seven cultivars suggested that the winter cultivars clustered together and stood out from the spring cultivars.

DREM analysis of this additional dataset, using the TF-gene interactions information described above and in Additional file 2, revealed that these three varieties are controlled by the same set of CBF and non-CBF TFs at the onset of cold stress. Additional file 2: Figure S11 shows the regulatory map of crown and leaf of the winter Harnesk. The highest path of this map is controlled by: CBF1, CBF2, CBFIIId-12, CBFIIId-B19, CBFIVa-A2, ICE1, RAP2.3 and RAP2.12, which are the same TFs that were involved in the control of wM, wN, sM, and sN. This result suggests that genes that are controlled by these TFs are co-regulated and conserved across the 7 wheat cultivars.

## Discussion

Results obtained in this study revealed significant regulatory patterns associated with the wheat response to low temperatures. Our analysis also revealed new genes involved in differential response to cold between wheat cultivars.

### The CBF cold responsive pathway is conserved in wheat

It is well documented in the literature that the CBF pathway is activated upon cold stress and plays a central role in plant response to cold [1–21, 24]. In this study, we identified CBFIIId-12, CBFIIId-B19, CBFIVa-A2, CBF1 and CBF2 as positive regulators of cold-responsive genes. Conversely, CBFIIIc-B10, CBFIIIc-D3, TaCBF6, CBFII-5.2 and TmCBF7 were identified to be negatively regulated during cold stress.

The linear model inferred that ICE1and ICE2 positively regulate CBFIIId-12, CBFIIId-B19, CBFIVa-A2, CBF1 and CBF2 and may play redundant roles. Interestingly, the dynamic regulatory map detected that these events (positive regulation) take place right at the onset of cold stress across all the four cultivars (Fig. 5 and Additional file 2: Figures S1, S2 and S3). These observations suggest that ICE1 and ICE2 are induced early by cold exposure, and subsequently regulate the expression of CBF TFs. This is consistent with experimental observations in Arabidopsis [51]. The linear model revealed negative regulation of ICE1 on MYB15. In *Arabidopsis*, ICE1 appears to negatively regulate the expression of MYB15, and MYB15 suppresses CBFs [6]. The ICE1-MYB15 axis thus seems to play an important role in regulating CBF expression levels during cold acclimation [6]. It is reasonable to infer that ICE1 may upregulate CBFs by suppressing MYB15 (Figs. 3 and 4d).

To complete this picture, several CBF TFs in the linear model are shown to regulate genes that are linked to cold shock, low temperature response, ice recrystallization, cold-regulated proteins, lipids and FA metabolism (Fig. 2 and Additional file 1). Interestingly, GO analysis of genes in the paths associated with these TFs (highest ones after 35 days, for example) also revealed an association with these terms. These cold-responsive genes are known to encode a diverse array of proteins such as enzymes involved in cell respiration and in the metabolism of carbohydrates, lipids, phenylpropanoids and antioxidants. Furthermore, they also encode molecular chaperones, antifreeze proteins, and other proteins with a potential function in tolerance to dehydration caused by cold [1].

Association among ICE, MYB and CBF TFs appeared at the onset of cold shock (first split of all four dynamic regulatory maps, Fig. 5 and Additional file 2: Figures S1, S2 and S3). Through the linear model, regulatory relationships were inferred both among the CBF TFs themselves and between the CBF TFs and cold responsive genes. Like in other plant species, the CBF cold response pathway was found to be conserved in the wheat cultivars analyzed in this study. The CBF regulatory network appears to play a pivotal role in wheat during cold stress.

### Several classes of TFs besides CBFs also play important role in cold acclimation

The linear model identified several interactions between non-CBF TFs. The association of these non-CBF TFs with the first split of the dynamic regulatory map suggests that other classes of TFs, besides CBFs, may also play important roles in cold acclimation. These TFs include MYB1, MYB4, ESK1, ELO2/FEN, HOS3, RR6, RR2, PRR1, WRKY1, WRKY48, NF-YA6, NF-YA5, EIN4, EIN3, A20/AN1-like, SWI/SNF, MCB2 and MYBAS1. Using the plant REACTOME database [41] to analyze these TFs and TGs reveal that they belong, for the most part, to the gibberellin (GA), jasmonic (JA), ethylene (ETH), cytokine (CK), or abscisic acid (ABA) pathways, which are known to be activated during cold exposure and to play a major role during cold acclimation in Arabidopsis [6, 52, 53]. EIN3, for example, is activated upon cold stress by ethylene and contributes to the down-regulation of CBF expression and type-A RR genes [52].

In Arabidopsis, the *eskimo1* (*esk1*) mutant accumulates high levels of *proline* and exhibits freezing tolerance when exposed to freezing temperature [54]. Transcriptome comparison of CBF2-overexpressing plants and *esk1* mutants showed that ESK1 and CBF2 regulate different sets of genes [54]. This was also confirmed in the present study. The dynamic regulatory map in Fig. 5 shows the regulation of a different set of genes by ESK1 and CBFs after the second day (2^nd^ highest split of the map) of cold treatment. In addition, the linear model and the regulatory map showed that ESK1 is associated with the regulation of nearly 100 genes that were differentially expressed in response to cold stress. MYB4 was also shown to be associated with a majority of these 100 genes. In rice, MYB4 has been shown to be induced by cold and to transactivate the expression of COR genes (RD29A, COR15a and PAL2) [55]. In our analysis, COR genes were found to be co-expressed with the TGs of MYB4.

We identified a group of RR and pseudo-RR TFs that appear to be cold-sensitive and may also play a role in wheat under cold stress. This group of TFs includes RR6, RR2 and PRR1, and may either be activated directly by cold or by the cytokine pathways [56, 57]. Two WRKY TFs, WRKY1 and WRKY48, were also identified to be involved in cold stress in this study. WRKY TFs have previously been reported to be involved in various physiological programs and in response to pathogens [58]. It has also been reported that WRKY transcription factors are involved in cold hardening of wheat [59]. Several stress-related effector genes were co-regulated with the WRKY TFs.

It is interesting to note that several TFs that were active at the onset of cold stress had previously been linked to cold stress not only in wheat but also in other plant species. However, there were also other TFs (WRKY1, WRKY48, RAP2.12 and RAP2.3) for which we could not find any literature evidence of involvement in cold stress. For example, RAP2.12 has recently been identified as an activator of the alcohol dehydrogenase 1 (ADH1) gene. It has also been shown that RAP2.12 and its homologues RAP2.2 and RAP2.3 act redundantly in multiple stress responses [60]. The co-expression of RAP2.12 and RAP2.3 with CBF1, CBF2, CBFIIId-12, CBFIII-B19 and CBFIVa-A2 suggests that RAP2.12 and RAP2.3 are regulated by CBFs and may play a role in cold stress. Further analysis of these TFs may shed new light into the regulation of cold-responsive genes in wheat.

### Adaptation of fatty acid and lipid metabolism to cold stress

The lipid composition in membranes is known to play a pivotal role in the plant response to cold stress. Alteration in membrane lipid composition allows plants to survive in the cold, whereas FA composition in membrane is the key factor determining fluidity of the cell membrane. Saturated FAs ensure more rigidness as they favor hydrophobic interactions whereas unsaturated FAs increase the fluidity of the cell membrane [61]. This information correlates well with our analysis. For example, fatty acid desaturase 7 (FAD7, FADD, Ta.24254.1.S1_a_at), which is involved in the desaturation of linoleic acid (18:2) to alpha-linolenic acid (18:3), decreased significantly after cold exposure (Additional file 2: Figure S5). Indeed, it has been observed that the saturated to unsaturated fatty acid ratio decreases under cold stress in cold-tolerant plant species. An alteration of unsaturated fatty acids (UFA) levels has also been reported in cold-stressed plants. Along with this, a high abundance of 18:3 is observed as compared to 18:1 and 18:2 linoleic acids, thus indicating the importance of FAs elongation and desaturation in tolerance against cold stress [62]. In Arabidopsis, the FAD7 gene encodes a plastid omega-3 FA desaturase that catalyzes the desaturation of dienoic FAs in membrane lipids [62, 63]. FAD7 may play a similar role in wheat.

It is interesting to see that FAD7, in its decreased expression, is co-expressed with three other lipid metabolism-related genes that also play important roles in abiotic stresses: DGK5 and SQD2 (Ta.3876.1.A1_at and Ta.24785.1.A1_at, Additional file 2: Figure S5). SDQ2, the sulfolipid sulfoquinovosyldiacylglycerol is one of the three nonphosphorous glycolipids that provide the bulk of the structural lipids in photosynthetic membranes of seed plants [64]. DGK5 is involved in the accumulation of phosphatidic acid during cold stress [65].

High correlations among FA-regulated genes (Additional file 2: Figures S4 and S5) suggest the possibility that these genes are co-regulated by the same group of TFs. Indeed, the linear model inferred that FA genes are regulated by a cascade of interactions between HOS3, ELO2, ESK1, MYB4 and ICE2. ELO2 has been shown to be involved in the synthesis of VLCFAs, which are essential precursors for sphingolipids and ceramides that play key roles during cold stress and in the control of stomatal behavior [66]. Furthermore, HOS3 has been shown to inhibit ABA-mediated stress responses and be involved in the VLCFA pathway and products as control points for several aspects of abiotic stress signaling and responses [66]. Interestingly, among the targets of ELO2 and HOS3, we have found several FA genes, including FAR1, FAR4 and FAR5, which are involved in the synthesis of VLCFA [43].

### Differences in spring and winter wheat

Based on the results obtained by the OPTricluster algorithm, we hypothesized that differences among same genes in terms of fold change across cultivars, and most importantly, delay in terms of activation or repression of the same genes across cultivars may hold or play key roles in the phenotypic differences among winter and spring wheat varieties, and their adaptation to LT stress, cold shock and cold acclimation.

Indeed, it has long been argued that wheat varieties can be divided on the basis of whether they require an extended period of cold to flower (vernalization). Varieties that have a requirement for vernalization also tend to be winter hardy and are able to withstand quite extreme subzero temperatures [3, 4]. Divergent patterns describing the expression profile of four MADX-box genes (Additional file 2: Figure S8) for example, show co-expression similarities among winter varieties and among spring varieties. The expression levels of three MADS-box TFs, MADS2 (TaAffx.120063.1.S1_at), TaAGL29 (Ta.3583.1.A1_at), and MADS-box transcription factor (Ta.7594.1.A1_s_at), significantly increased from the beginning and throughout the experiment in the two spring cultivars (**sN** and **sM**) and their activation appeared to be delayed in the two winter cultivars (**wM** and **wN**). One MADX-box gene (TaAffx.143995.17.S1_s_at, VRN-A1) appears to have an opposite expression pattern, increased earlier in the winter cultivars (**wM** and **wN**) but later in the spring cultivars. This suggests that TaAffx.143995.17.S1_s_at perhaps plays an important regulatory role in activating the wheat metabolic machinery against cold stress. It has indeed been previously hypothesized that some Cor/Lea genes may be co-regulated by the vernalization (VRN) genes during cold acclimation and these VRN genes may also control the expression of CBFs [46]. They may also represent target genes useful in breeding programs. TaVRT-1 (TA.142.1.S1_AT), VRN-A1 (TaAffx.143995.17.S1_s_at) and VRN-B1 (Ta.30607.1.A1_at), also identified in this study, have been previously shown to be functional markers linked with agronomic traits in wheat [3, 24, 47]. The fact that spring varieties had more differentially expressed genes compared to the winter ones may also find their answers in the delay reaction of certain TFs/genes across wheat varieties.

## Conclusions

In this study, we combined three computational techniques to reconstruct dynamic regulatory events in wheat following cold stress, and to study similarities and differences among seven wheat cultivars, not only based on their expression profiles, but also on the timing of activation. Our three-way analysis showed that the CBFs pathways is conserved and played a pivotal role in wheat under cold stress. CBF TFs accumulated at onset of cold stress and regulated the expression of cold responsive genes in a transient way. Beside CBF TFs, other TFs were also involved in the regulation of cold responsive genes in CBF-independent pathways. We identified several patterns that were conserved across wheat cultivars. New knowledge was inferred from these conserved patterns. We assigned novel cold-related roles to 35 wheat genes, uncovered novel TF-gene interactions, and identified 127 genes representing known and novel candidate targets for genomic assisted breeding of cold-resistant wheat. This study provides novel computational insights into the underlying mechanisms that regulate the expression of cold-responsive genes in wheat, the timing of these regulatory events and the complexity of the mechanisms that determine LT adaptation in spring and winter wheat.

## Additional files

Additional file 1: Selected and significant transcription factor gene interactions data inferred by the linear model and used as input to the DREM algorithm. The first column corresponds to the source (transcription factor). The second column corresponds to the target (gene or transcription factor). The third column corresponds to the type of interactions, positive (+1) or negative (-1) interactions. (TXT 26 kb)
Additional file 2: This file contains additional **Figures S1, S2, S3, S4, S5, S6, S7, S8, S9, S10 **and **S1**, and **Tables S1, S2, S3** and **S4** mentioned in the manuscript (PDF file). (PDF 1879 kb)

## Abbreviations

3D: Three dimensions
CBF: C-repeat Binding Factors
ChIP-chip: Chromatin immunoprecipitation
DREM: Dynamic Regulatory Event Miner
GEO: Gene Expression Omnibus
GO: Gene Ontology
GOAL: Gene Ontology AnaLyzer
IOHMMs: Input-output hidden Markov models
LT: Low temperature
OPTricluster: Order Preserving Triclustering Algorithm
PlantTFDB: Plant Transcription Factor Database
PLEXdb: Gene expression ressources for plants and plants pathogens
POB: Plant Orthology Browser
sM: Spring Manitou
sN: Spring Norstar
sP: Spring Paragon
TF: Transcription factors
TFA: Transcription factor activity
wH: Winter Harnesk
wM: Winter Manitou
wN: Winter Norstar
wS: Winter Solstice
