# Supplementary material for: Bioinformatics identification of new targets for improving low temperature stress tolerance in spring and winter wheat
Source: BMC Bioinformatics. 2017 Mar 16;18:174. doi: 10.1186/s12859-017-1596-x (PMC5356398; doi:10.1186/s12859-017-1596-x)
Supplement: Additional file 2: — This file contains additional Figures S1, S2, S3, S4, S5, S6, S7, S8, S9, S10 and S1, and Tables S1, S2, S3 and S4 mentioned in the manuscript (PDF file). (PDF 1879 kb) [file 12859_2017_1596_MOESM2_ESM.pdf]

Bioinformatics identification of new targets for improving low temperature stress tolerance in spring and winter wheat

Alain B. Tchagang[1$], François Fauteux[1], Dan Tulpan[2], and Youlian Pan[1]

[1]Information and Communications Technologies, National Research Council Canada, Ottawa, Ontario, K1A 0R6, Canada

[2]Information and Communications Technologies, National Research Council Canada, Moncton, New Brunswick, E1A 7R1, Canada

Additional file 2

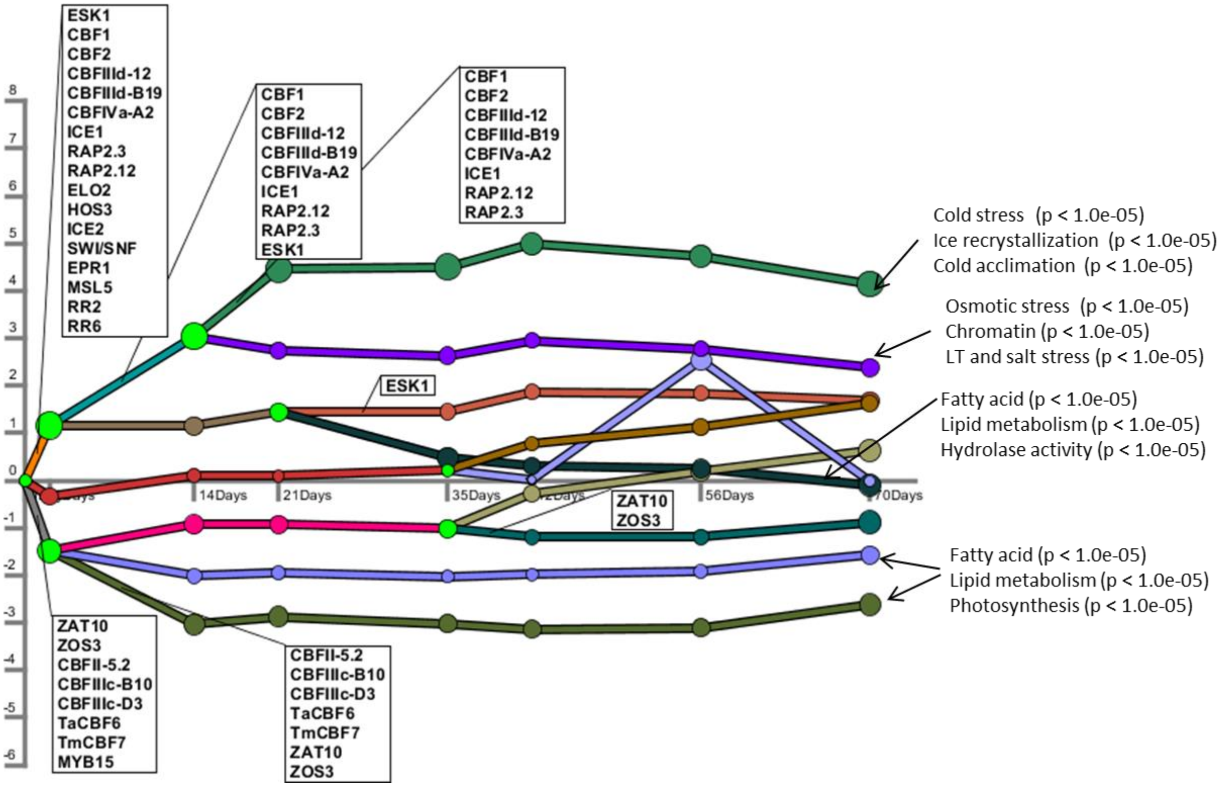

Figure S1: Dynamic regulatory map of winter Norstar. The x-axis is the time-points in days. The y-axis is the gene expression levels. This map contains 10 paths. Each path corresponds to the mean of the fold change of expression level of the genes that belong to it, relative to day 0. The size of nodes corresponds to standard deviations of the fold changes. This map is obtained using the TF-gene interactions (**Figure 2**, **Additional file 1**) and the gene expression data of the winter Norstar as input to the DREM algorithm, using a TF split cutoff score of 0.005.

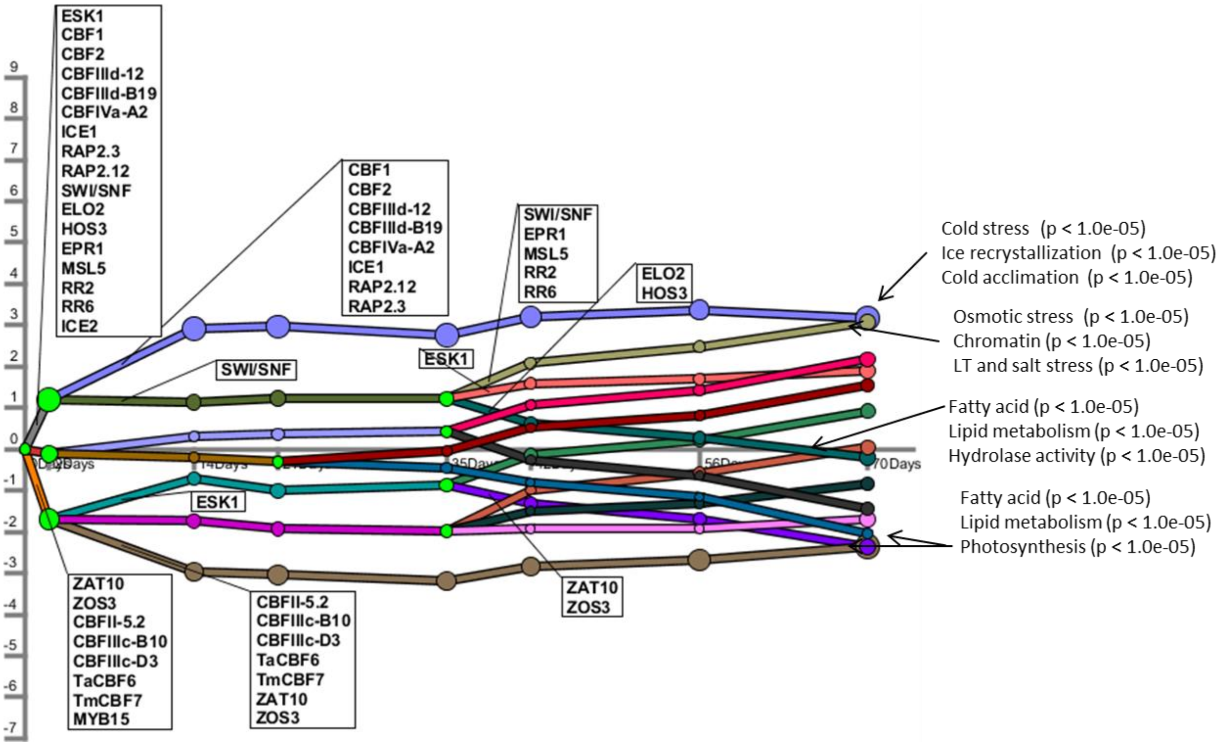

Figure S2: Dynamic regulatory map of spring Manitou. The x-axis is the time-points in days. The y-axis is the gene expression levels. This map contains 14 paths. Each path corresponds to the mean of the fold change of expression level of the genes that belong to it, relative to day 0. The size of nodes corresponds to standard deviations of the fold changes. This map is obtained using the TF-gene interactions (**Figure 2**, **Additional file 1**) and the gene expression data of the spring Manitou as input to the DREM algorithm, using a TF split cutoff score of 0.005.

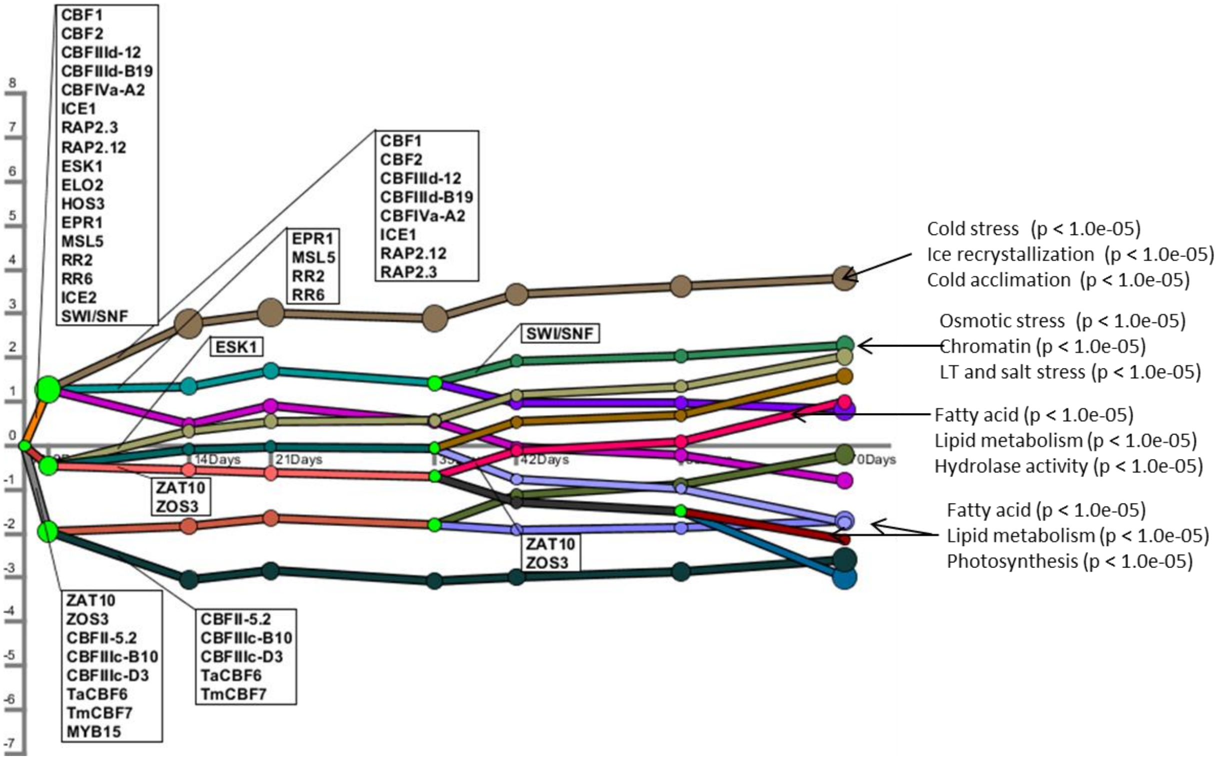

Figure S3: Dynamic regulatory map of spring Norstar. The x-axis is the time-points in days. The y-axis is the gene expression levels. This map contains 13 paths. Each path corresponds to the mean of the fold change of expression level of the genes that belong to it, relative to day 0. The size of nodes corresponds to standard deviations of the fold changes. This map is obtained using the TF-gene interactions (**Figure 2**, Additional **file 1**) and the gene expression data of the spring Norstar as input to the DREM algorithm, using a TF split cutoff score of 0.005.

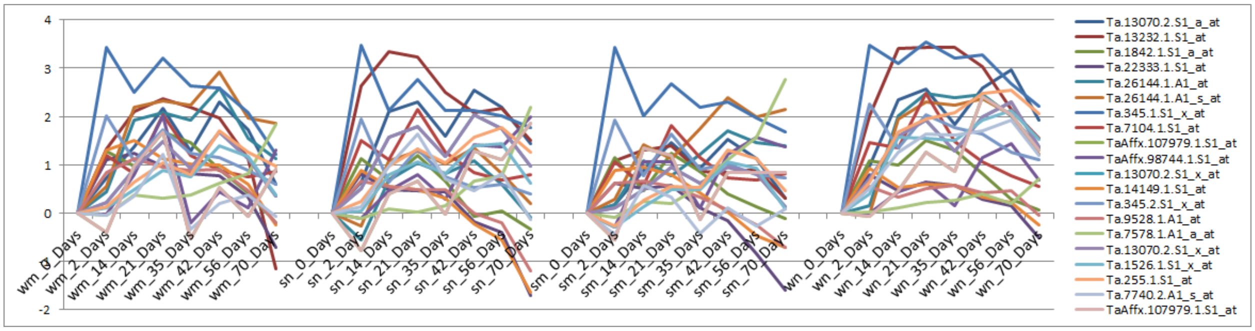

Figure S4: Twenty up-regulated probes corresponding to eighteen unique genes with similar behavior across the four cultivars wM, sM, wN and sN linked to fatty acid. The y-axis is the fold change of gene expression level relative to day 0. Each line corresponds to the expression profile of a gene across the time series. This cluster was obtained using the OPTricluster algorithm and the 3D time series gene expression data of the four wheat cultivars. Table below lists the set of genes in **Figure S4** and their Affymetrix description.

Probeset	Reference Description
Ta.13070.2.S1_a_at	LTPL10 - Protease inhibitor/seed storage/LTP family protein precursor, expressed
Ta.13232.1.S1_at	Symbols: SMO1-2, ATSMO1, ATSMO1-2, SMO1 | sterol C4-methyl oxidase 1-2 | chr4:11955124-11956695 REVERSE LENGTH=300
Ta.1842.1.S1_a_at	Phosphoethanolamine methyltransferase [Triticum aestivum (Wheat)]
Ta.22333.1.S1_at	Symbols: FAR4 | fatty acid reductase 4 | chr3:16124079-16127769 FORWARD LENGTH=494
Ta.26144.1.A1_at	Symbols: FAR1 | fatty acid reductase 1 | chr5:7470541-7473916 FORWARD LENGTH=492
Ta.345.1.S1_x_at	Symbols: | alpha/beta-Hydrolases superfamily protein | chr1:27629266-27632486 FORWARD LENGTH=464
Ta.345.1.S1_x_at	Monoglyceride lipase isoform 2-like [Oryza sativa (japonica cultivar-group)]
Ta.7104.1.S1_at	putative phosphoethanolamine methyltransferase [Oryza sativa (japonica cultivar-group)]
TaAffx.107979.1.S1_at	Sterol 14-demethylase n=2 Tax=Arabidopsis RepID=CP511_ARATH
TaAffx.98744.1.S1_at	Obtusifoliol 14-alpha demethylase n=9 Tax=Poaceae RepID=CP51_SORBI
Ta.13070.2.S1_x_at	LTPL10 - Protease inhibitor/seed storage/LTP family protein precursor, expressed
Ta.14149.1.S1_at	Symbols: FAR5 | fatty acid reductase 5 | chr3:16138060-16141409 FORWARD LENGTH=497
Ta.345.2.S1_x_at	Symbols: | alpha/beta-Hydrolases superfamily protein | chr1:27629266-27632486 FORWARD LENGTH=464
Ta.9528.1.A1_at	Symbols: FAR4 | fatty acid reductase 4 | chr3:16124079-16127769 FORWARD LENGTH=494
Ta.7578.1.A1_a_at	Symbols: ACS | acetyl-CoA synthetase | chr5:14534961-14540296 REVERSE LENGTH=744
Ta.1526.1.S1_x_at	Type 1 non specific lipid transfer protein precursor [Triticum aestivum (Wheat)]
Ta.255.1.S1_at	Lipid transfer protein precursor [Triticum aestivum (Wheat)]
Ta.7740.2.A1_s_at	Putative lipase [Oryza sativa (japonica cultivar-group)]

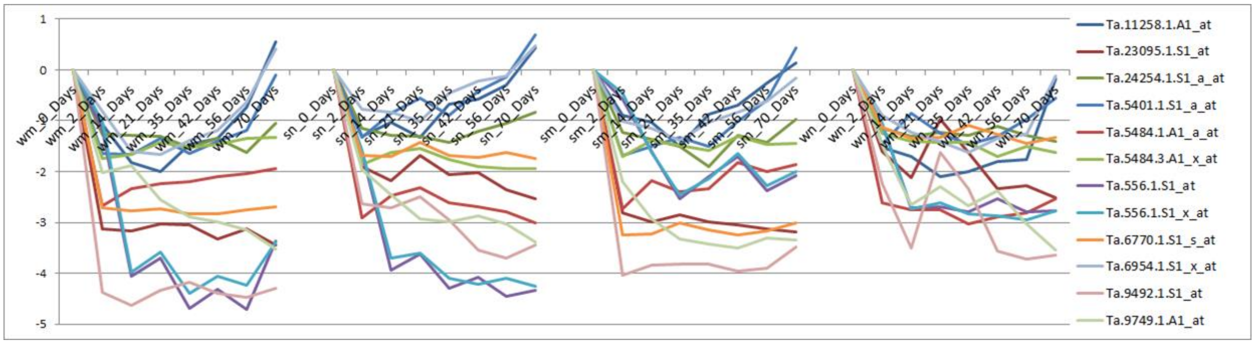

Figure S5: Twelve down-regulated probes IDs corresponding to ten unique genes with similar behavior across the four cultivars (wM, sM, wN and sN), that are linked to fatty acid. The y-axis is the fold change of gene expression level relative to day 0. Each line corresponds to the expression profile of a gene across the time series. This cluster was obtained using the OPTricluster algorithm and the 3D time series gene expression data of the four wheat cultivars. Table below lists the set of genes in **Figure S5** and their Affymetrix description.

**Probeset**	**Reference Description**
Ta.11258.1.A1_at	phosphotransferase, putative, expressed
Ta.23095.1.S1_at	glycerophosphoryl diester phosphodiesterase family protein, putative, expressed
Ta.24254.1.S1_a_at	| Symbols: FAD7, FADD | fatty acid desaturase 7 | chr3:3499959-3502126 FORWARD LENGTH=447
Ta.24254.1.S1_a_at	Omega-3 fatty acid desaturase, chloroplastic n=1 Tax=Sesamum indicum RepID=FAD3C_SESIN
Ta.24785.1.A1_at	glycosyl transferase, group 1 domain containing protein, expressed
Ta.29440.1.S1_at	phosphotransferase, putative, expressed
Ta.3876.1.A1_at	| Symbols: DGK5, ATDGK5 | diacylglycerol kinase 5 | chr2:8989794-8992798 REVERSE LENGTH=492
Ta.5401.1.S1_a_at	| Symbols: | alpha/beta-Hydrolases superfamily protein | chr2:16460442-16462872 FORWARD LENGTH=318
Ta.5401.1.S1_a_at	monoglyceride lipase, putative
Ta.5484.1.A1_a_at	flavonol sulfotransferase-like [Zea mays]
Ta.5484.3.A1_x_at	flavonol sulfotransferase-like [Zea mays]
Ta.556.1.S1_at	lipase, putative, expressed
Ta.6770.1.S1_s_at	| Symbols: | PLC-like phosphodiesterases superfamily protein | chr5:16441858-16443835 FORWARD LENGTH=375
Ta.6954.1.S1_x_at	Putative choline kinase CK2 [Oryza sativa (japonica cultivar-group)]
Ta.9749.1.A1_at	| Symbols: | alpha/beta-Hydrolases superfamily protein | chr2:13256738-13258174 REVERSE LENGTH=415

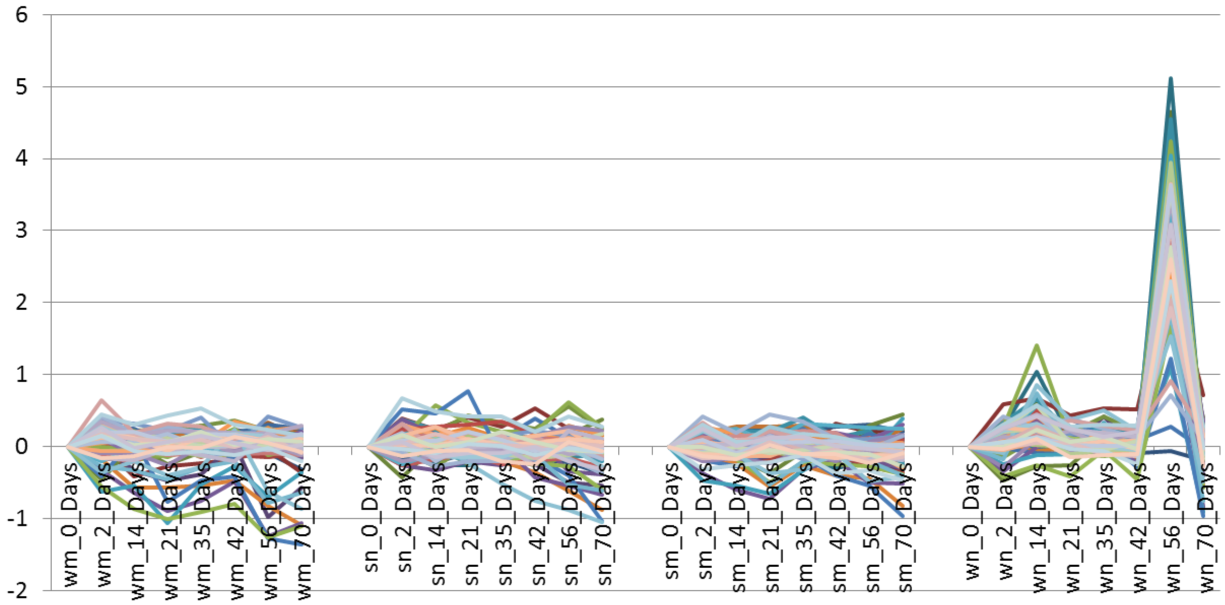

Figure S6: Example of genes with dissimilar patterns across the experimental time points. The y-axis is the fold change of gene expression level relative to day 0. Each line corresponds to the expression profile of a gene across the time series. This cluster was obtained using the OPTricluster algorithm and the 3D time series gene expression data of the four wheat cultivars.

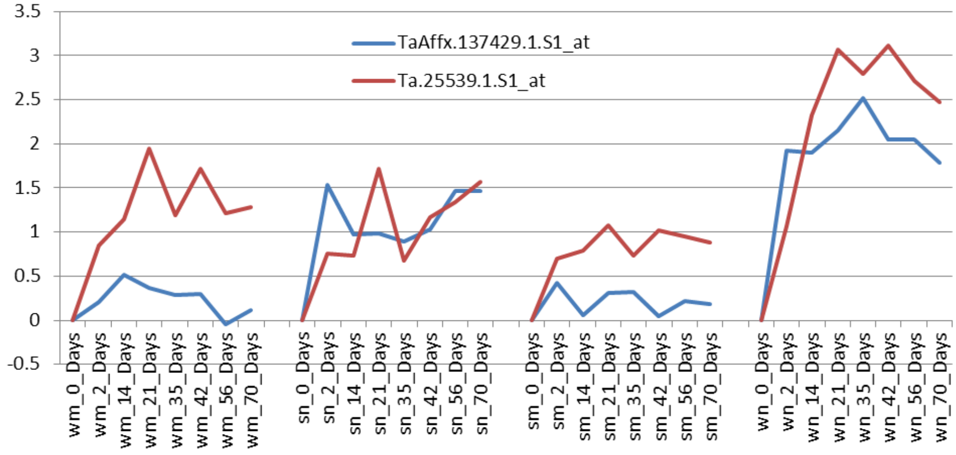

Figure S7: Example of genes with dissimilar patterns across the experimental time points. The y-axis is the fold change of gene expression level relative to day 0. Each line corresponds to the expression profile of a gene across the time series. This cluster was obtained using the OPTricluster algorithm and the 3D time series gene expression data of the four wheat cultivars. TaAffx.137429.1.S1_at (similar to UniRef100_O65216 Cluster: COR39; n=1; Triticum aestivum|Rep: COR39 - Triticum aestivum) and Ta.25539.1.S1_at (Cold-regulated protein BLT14 n=1 Tax=Hordeum vulgare RepID=CR14_HORV).

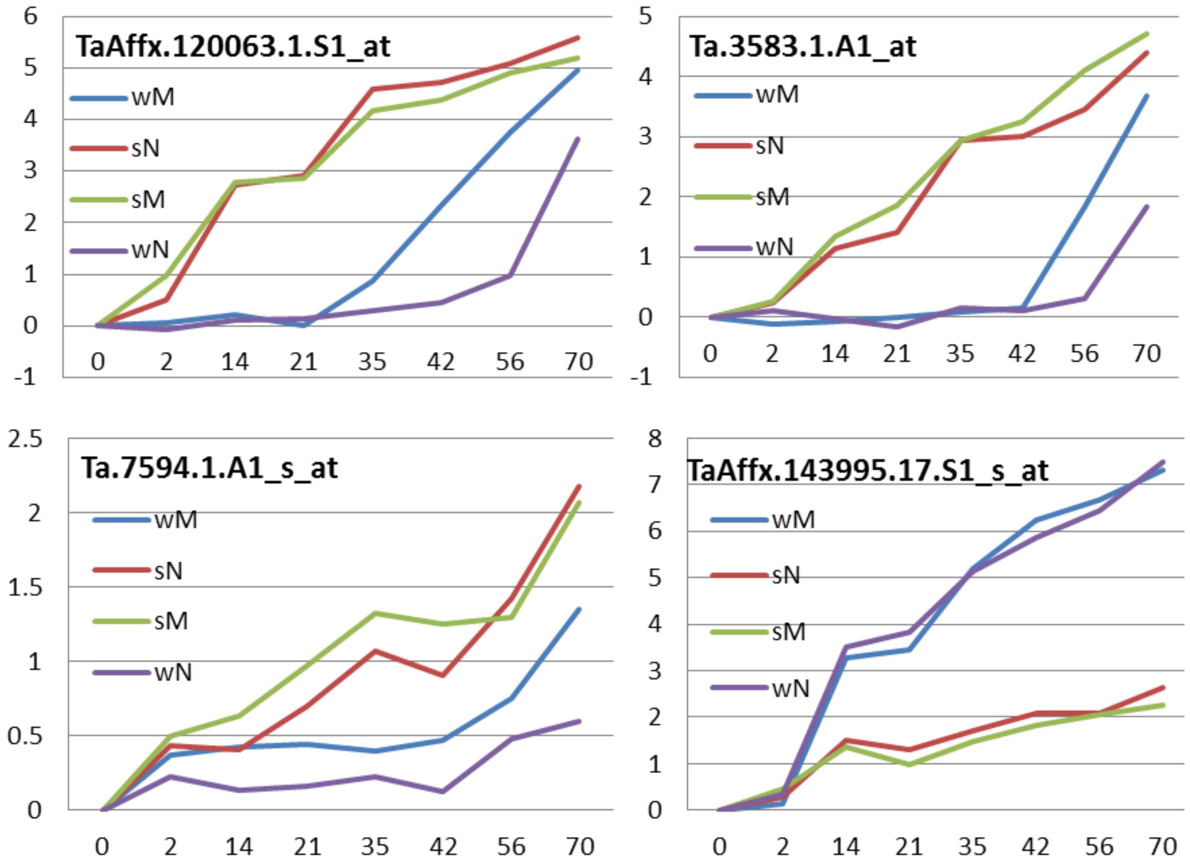

Figure S8: Example of four genes with dissimilar patterns across the experimental time points. The y-axis is the fold change of gene expression level relative to day 0. Each line corresponds to the expression profile of a gene across the time series. This cluster was obtained using the OPTricluster algorithm and the 3D time series gene expression data of the four wheat cultivars. TaAffx.120063.1.S1_at (MADS2), Ta.3583.1.A1_at (TaAGL29), Ta.7594.1.A1_s_at (MADS-box transcriptional factor), and TaAffx.143995.17.S1_s_at (VRN-A1).

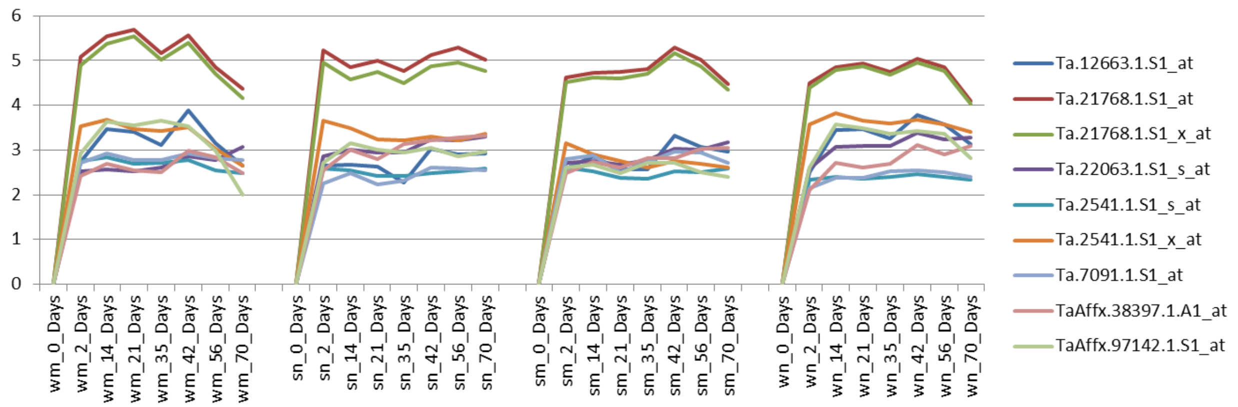

Figure S9: Example of six genes with similar behavior across the experimental time points (conserved patterns). The y-axis is the fold change of gene expression level relative to day 0. Each line corresponds to the expression profile of a gene across the time series. This cluster was obtained using the OPTricluster algorithm and the 3D time series gene expression data of the four wheat cultivars. Table below lists the set of genes in **Figure S9** and their Affymetrix description.

**Probeset**	**Reference Description**
Ta.12663.1.S1_at	UniRef100_Q56B90 Cluster: Ice recrystallization inhibition protein 1 precursor; n=1; Triticum aestivum
Ta.21768.1.S1_at	similar to UniRef100_Q56B90 Cluster: Ice recrystallization inhibition protein 1 precursor; n=1;
Ta.21768.1.S1_x_at	similar to UniRef100_Q56B90 Cluster: Ice recrystallization inhibition protein 1 precursor; n=1; Triticum aestivum
Ta.22063.1.S1_s_at	no hit TC421941,
Ta.2541.1.S1_s_at	homologue to UniRef100_P93614 Cluster: Cold acclimation protein WCOR615; n=1; Triticum aestivum
Ta.2541.1.S1_x_at	homologue to UniRef100_P93614 Cluster: Cold acclimation protein WCOR615; n=1;
Ta.7091.1.S1_at	Symbols: P5CS2 | delta 1-pyrroline-5-carboxylate synthase 2
TaAffx.38397.1.A1_at	weakly similar to UniRef100_Q10QT4 Cluster: Expressed protein;
TaAffx.97142.1.S1_at	No hit TC385582, weakly similar to UniRef100_Q2XSD8 Cluster: Seed maturation protein; n=1; Glycine tomentella

Gene OscilloScope Results

bars and the values represent CV in % (Coeff. of Variation of the treatment means in the corresponding expt.)
CLICK the cell for more details (expression graph of the probe set in an experiment).
Based on data normalized by **RMA** from **Wheat** genechip (using **3 probe sets** from **your input**).

Expts.	No of treats.	Ta.123.1.S1_x_at	Ta.124.1.S1_x_at	Ta.145.1.A1_x_at	Experiment Name
TA1	2	0	0	0	TA1: Rehybridization of Wheat and Barley cRNAs to the Affymetrix Wheat Genome Arrays
TA2	2	0	0	0	TA2: Wheat GeneChip Test
TA3	13	5	26	7	TA3: Transcription patterns during wheat development (Mirrors BB3)
TA5	39	4	6	5	TA5: Expression Profile Mapping - 78 chip experiment final
TA6	2	5	2	2	TA6: Expression Profile Mapping - 78 chip experiment parents
TA7	2	0	12	4	TA7: Expression Profile Mapping - Location 2 - parental genotypes
TA8	36	2	4	4	TA8: Expression Profile Mapping - Location 2 - mapping population
TA9	16	8	10	23	TA9: Wheat Yr5 isolines treated with P.s. tritici PST-100 (06-194)
TA10	5	5	2	7	TA10: Mapping translocation breakpoints using a wheat microarray
TA11	12	4	10	5	TA11: Wheat Yr39 and yr39 (Alpowa) genotypes treated with P.s. tritici PST-78
TA20	8	4	4	5	TA20: Transcriptome analysis of a wheat NIL pair carrying FHB resistant and susceptible alleles
TA22	4	25	24	26	TA22: Freeze Resistance basis of winter wheat mutant lines
TA23	9	5	14	7	TA23: Drought stress in Wheat at grain filling stage
TA24	4	1	5	2	TA24: Transcription profiling wheat responses to adapted and non-adapted isolates of the blast fungus, Magnaporthe
TA25	3	1	3	1	TA25: Transcription profiling of wheat interacting with incompatible and compatible yellow rust in a Yr1-containing genotype
TA26	3	1	14	1	TA26: Transcriptome analysis of two Yr5 deletion mutants
TA27	7	4	4	4	TA27: Microarray expression analysis of meiosis and microsporogenesis in hexaploid bread wheat
TA28	38	3	6	4	TA28: Wheat expression level polymorphism study parentals and progenies from SB location
TA29	12	3	18	5	TA29: Expression data from rust or mock inoculated, fully expanded flag leaf halves
TA30	8	3	3	4	TA30: Genotypic differences in water soluble carbohydrate metabolism in stem
TA31	2	0	6	0	TA31: Gene expression analysis of the wheat response to infection by Fusarium pseudograminearum
TA32	12	7	12	7	TA32: Transcript profiling of Lr1- and Lr34-mediated leaf rust resistance in wheat
TA33	4	8	2	5	TA33: Wild emmer wheat comparison of drought resistant vs. susceptible genotypes under terminal drought
TA34	4	3	3	3	TA34: Transcriptomic analysis of the effect of silicon on wheat plants infected or uninfected with powdery mildew
TA35	8	7	13	9	TA35: RNA profiling of Fusarium head blight-resistant wheat addition lines containing the Thinopyrum elongatum chromosome 7E
TA36	6	2	2	5	TA36: Pleiotropic expression of endogenous genes upon fungus infection of wheat plants containing anti-fungal transgenes
TA37	3	4	17	14	TA37: Metabolism-based resistance to herbicides in black-grass
TA38	5	5	8	9	TA38: affy_seed_kinetic_wheat-Transcriptomic wheat seed
TA39	4	8	7	4	TA39: Expression data in wheat (T. aestivum L.) near isogenic lines in response to powdery mildew infection
TA40	48	3	6	6	TA40: Differential transcriptome analyses of three wheat genotypes in their response to Fusarium Head Blight and trichothecenes
TA41	30	4	8	10	TA41: Response to Fusarium in near-isogenic lines harboring resistance QTLs Fhb1 and Qfhs.ifa-5A
TA42	32	20	16	17	TA42: Transcriptome profiling and expression analyses of genes critical to wheat adaptation to low temperature
TA43	4	38	41	29	TA43: Expression data from Triticum aestivum L. roots
TA44	8	7	10	10	TA44: Transcriptome profiling of reproductive stage flag leaves of wheat from drought susceptible parent WL711, drought tolerant pare
TA45	9	10	27	6	TA45: Global transgenerational gene expression dynamics in two nascent allohexaploid wheat lines analogous in genome constitution

Figure S10: Validation of three genes Ta.123.1.S1_x_at (Cold acclimation protein WCOR80), Ta.124.1.S1_x_at (Cold-shock protein CS120) and Ta.145.1.A1_x_at (Cold shock protein CS66) identified in this study and not in [reference 3], using the Gene Oscilloscope of the PLEXdb (http://www.plexdb.org/modules/tools/genoscope/genoscope.php). This result shows that these three probe sets have coefficient of variations of 20, 16, and 17 respectively, for the row TA42 which corresponds to the wheat datasets tested in this study.

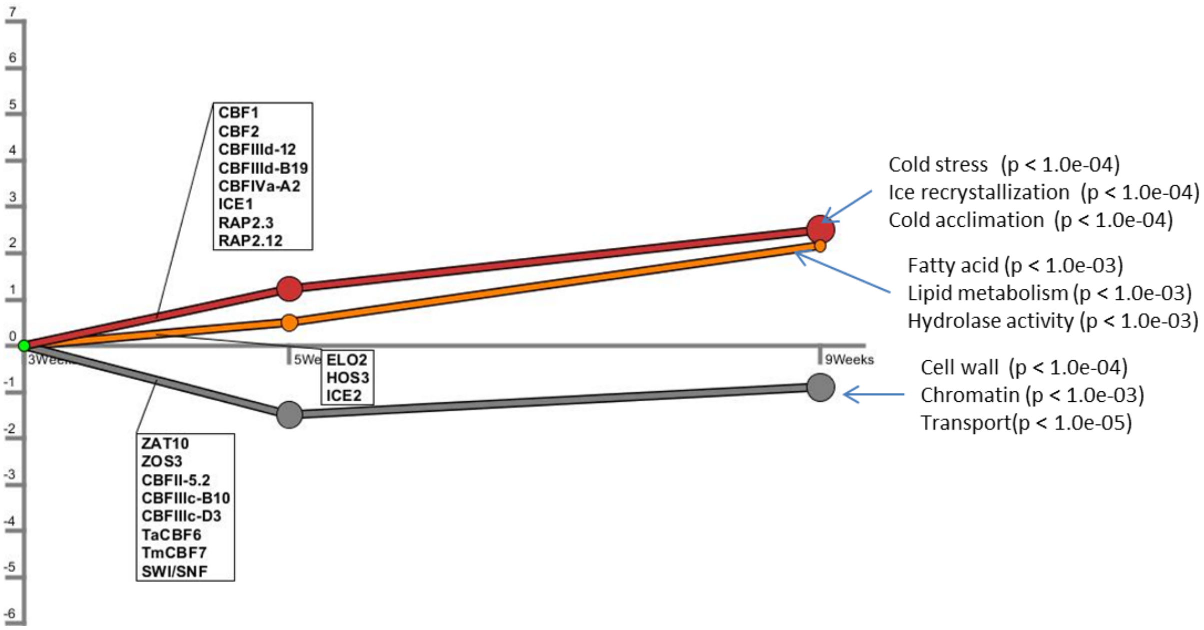

Figure S11: Dynamic regulatory map of crown and leaf Harnesk. The x-axis is the time-points in days. The y-axis is the fold change of gene expression levels. This map contains 3 paths. Each path corresponds to the mean of the expression level of the genes that belong to it. The size of nodes corresponds to standard deviations. This map is obtained using the TF-gene interactions (**Figure 2**, **Additional file 1**) and the gene expression data of the wheat Harnesk cultivar as input to the DREM algorithm as input to the DREM algorithm, using a TF split cutoff score of 0.005.

Table S1: List of conserved and upregulated genes in the four genotypes. This cluster of genes was obtained using the 3D time series gene expression data of the four wheat cultivars and the OPTricluster algorithm. Numbers in column 3-6 represent the fold change between the min and the max of the expression level across the experimental time points.

Probe ID	Description	wM	sN	sM	wN
Ta.10319.1.A1_s_at	Myb-like	4	3.8	5.2	1.7
Ta.10516.2.S1_at	Jacalin-like lectin domain	3.6	3.3	3.2	3.8
Ta.10857.1.A1_at	Cluster: Os10g0566400 protein	2.1	2.8	2	2.9
Ta.11120.1.S1_a_at	Cluster: Apoplastic invertase 1 (cell wall)	1.8	2.4	1.5	2.4
Ta.11120.1.S1_x_at	Cluster: Apoplastic invertase 1 (cell wall)	1.7	2.3	1.5	2.2
Ta.1138.1.S1_at	Cold-responsive LEA/RAB-related COR protein	5.7	5.5	4.9	6.9
Ta.1165.1.A1_at	Cold-responsive protein COR14a	7.9	7.6	7.7	7.5
Ta.123.1.S1_x_at	Cold acclimation protein WCOR80	5.5	6	4.3	6.8
Ta.124.1.S1_x_at	Cold-shock protein CS120 (Dehydrin)	5.1	4.7	4	4.7
Ta.12663.1.S1_at	Ice recrystallization inhibition protein 1 precursor	3.9	3	3.3	3.8
Ta.13134.1.A1_at	Immediate early protein ICP0	4.6	3.9	5	4.2
Ta.13183.1.S1_s_at	Cold regulated protein (wcor18)	3.4	4.1	2.7	5.1
Ta.13183.1.S1_x_at	Cold regulated protein (wcor18)	4.2	4.7	3.3	6.5
Ta.13193.1.S1_at	NADH-ubiquinone oxidoreductase	3.7	3.5	3.5	3.2
Ta.13232.1.S1_at	Sterol desaturase family protein (SMO1-2)	2.4	3.4	1.4	3.4
Ta.13239.1.S1_at	Pherophorin-C1 protein precursor	4.9	4.8	4.8	5.3
Ta.13595.1.A1_at	BQ169949	3	2.8	2.6	2.9
Ta.13784.1.S1_at	PTACR7 (BLT14.1 protein; n=1; Hordeum vul)	5.4	6.2	4.6	6.5
Ta.145.1.A1_x_at	Cold shock protein CS6	4.6	5	3.8	5.5
Ta.16280.1.S1_at	Vegetative cell wall protein	2.4	1.9	2	2.7
Ta.1722.1.S1_at	TC434908	4	2.7	4	2.5
Ta.18391.1.S1_at	CA635688	1.9	1.7	1.8	1.7
Ta.18574.1.A1_x_at	Ice recrystallization inhibition protein 1 precursor	4.3	4.4	3.4	5.2
Ta.18720.1.S1_a_at	Gamma-thionin (Defensin-like protein 1)	6.5	6	5.4	4.7
Ta.18720.1.S1_x_at	Gamma-thionin (Defensin-like protein 1)	6.6	6	6	4.7
Ta.18720.2.S1_x_at	Gamma-thionin (Defensin-like protein 1)	6.2	5.8	4.6	5
Ta.18720.3.S1_x_at	Gamma-thionin (Defensin-like protein 1)	5.9	5.2	5.6	4.3
Ta.19327.1.S1_at	Fe(III) dicitrate ABC transporter	3.6	3	3.8	2.8
Ta.21766.1.S1_at	Cold-regulated protein BLT14 (CR14)	4.1	4	4.3	3.9
Ta.21768.1.S1_at	Ice recrystallization inhibition protein 1 precursor	5.7	5.3	5.3	5
Ta.21768.1.S1_x_at	Ice recrystallization inhibition protein 1	5.5	5	5.2	5
Ta.22063.1.S1_s_at	TC421941	3.1	3.3	3.2	3.4
Ta.22764.1.S1_x_at	Low temperature and salt responsive protein	2.7	2.4	2.6	2.9
Ta.22766.1.S1_a_at	TC445245	6.9	7.3	8	7.3
Ta.22766.1.S1_at	TC445245	5.7	5.6	6.4	5.1
Ta.23419.1.S1_x_at	High molecular mass early light-inducible protein	2.6	3.5	2.8	2.8
Ta.23758.1.S1_x_at	Cold acclimation protein WCOR518	3.2	2.6	2.5	2.7
Ta.245.1.S1_at	Cold-responsive protein WCOR14c	6.1	7	5.9	7.4
Ta.24761.2.S1_at	TC376085	1.8	1.2	1.7	1.1
Ta.25026.1.S1_at	Dehydrin; (late embryogenesis abundant protein)	4.9	4.2	3.8	4.5
Ta.25077.1.A1_at	Ice recrystallization inhibition protein 2 precursor	2	1.6	1.7	2.5
Ta.2541.1.S1_s_at	Cold acclimation protein WCOR615	2.8	2.6	2.6	2.5
Ta.2541.1.S1_x_at	Cold acclimation protein WCOR615	3.7	3.7	3.2	3.8
Ta.25744.1.S1_at	TC416591 (Myb family), EPR1, REV7	3.7	3.7	3.4	4.4
Ta.25860.1.A1_at	Aquaporin PIP2	3.3	2.8	2.2	3.2
Ta.26928.1.S1_x_at	Fructan exohydrolase; glycosyl hydrolases	2.3	3.3	2	3.4
Ta.26928.3.S1_x_at	Fructan exohydrolase; glycosyl hydrolases	2.3	2.9	1.8	3.2
Ta.26929.2.S1_a_at	TC430899; E2f-associated phosphoprotein	2.8	3.3	2	3.5
Ta.2709.1.S1_s_at	Gamma-2-purothionin (Defensin-like protein)	4.9	3.9	5.1	3.4
Ta.27229.1.S1_at	Defensin precursor	2.7	0.9	2	1.1
Ta.27389.1.S1_at	Gamma-1-purothionin (Defensin-like protein 1)	5.3	5.1	4.3	4.8
Ta.27389.2.S1_x_at	Gamma-2-purothionin (Defensin-like protein 1)	5.3	4.5	5.4	3.4
Ta.27719.1.S1_at	5-oxoprolinase; n=1; Sphingomonas wittichii	5.2	5.2	5.1	5
Ta.27719.2.S1_x_at	TC448971	2.3	2.2	2.6	2
Ta.27725.1.S1_at	Low temperature-induced protein	2.8	2.6	2.8	2.3
Ta.2826.1.S1_at	Aquaporin PIP2	2.3	2.2	1.8	2.1
Ta.28273.1.S1_x_at	Low temperature and salt responsive protein	3	2.8	2.9	2.8
Ta.28369.1.S1_at	TC418413 Low Temperature and Salt	2.9	2.7	2.7	3.1
Ta.28537.1.S1_x_at	Defensin precursor	4.4	4.4	3.8	4.1
Ta.28917.1.S1_at	Cold acclimation protein WCOR518	3.2	2.6	2.8	2.7
Ta.28917.1.S1_x_at	Cold acclimation protein WCOR518 (LT seed	3.3	2.5	2.7	2.6

Ta.30336.1.S1_x_at	LEA D-11 dehydrin	6.2	6.2	4.8	5.9
Ta.30798.3.S1_at	C13 endopeptidase NP1 precursor	4.6	4.6	4.4	5.2
Ta.345.1.S1_at	TC385012 (alpha/beta-Hydrolases)	3.3	3.6	3.1	3.8
Ta.345.1.S1_x_at	TC385012 (alpha/beta-Hydrolases)	3.4	3.5	3.4	3.5
Ta.351.1.S1_at	Cold acclimation induced protein 2-1	1.5	1.8	1.5	2
Ta.3927.1.S1_at	TC378443 (thiamine biosynthesis protein thiC)	3.6	4.3	3.6	4.3
Ta.4035.2.S1_at	TC375124 (beta glucosidase 11)	2.3	2.8	2.1	3.1
Ta.4035.2.S1_x_at	TC375124 (beta glucosidase 11)	2.5	2.7	2.1	3.3
Ta.5888.1.S1_s_at	Polar amino acid ABC transporter (LEA)	3.7	3.8	1.4	5.6
Ta.613.1.S1_at	Group3 late embryogenesis abundant protein	4.2	4.5	3.9	5
Ta.6174.1.S1_at	Hydroxyproline-rich glycoprotein DZ-HRGP prec	1.2	1.8	1	3.3
Ta.7053.1.S1_at	TC427799	4.1	3.7	4	3.8
Ta.7091.1.S1_at	TC37493 (P5CS2)	2.9	2.6	3	2.6
Ta.759.1.S1_at	Cold acclimation protein WCOR413	3	2.5	2.5	2.7
Ta.7867.1.A1_at	Glycosyltransferase	3.4	2.8	3	1.6
Ta.7934.1.S1_x_at	Early nodulin 75-like protein	4.8	3.7	4.5	4.2
Ta.7934.2.S1_x_at	Early nodulin 75-like protein	6.2	5.3	4.6	5.5
Ta.7934.3.S1_at	HNH nuclease	4.8	3.3	4.3	3.9
Ta.8037.1.A1_at	Ice recrystallization inhibition protein 1 precurso	5.5	5.5	4.2	6.3
Ta.8085.1.S1_at	Expressed protein	3.9	4.5	4.2	4.5
Ta.819.1.A1_at	CA635685	0.7	0.7	0.4	0.6
Ta.865.2.A1_at	Expressed protein	4.6	4.6	4.2	5
Ta.9210.1.S1_a_at	Fe-superoxide dismutase (FSD2)	0.3	0.2	0.2	0.2
Ta.9481.2.S1_at	TC396927 (B12D protein)	3.2	1.6	2.7	1.5
Ta.9600.1.S1_x_at	Low molecular mass early light-inducible protein	2.7	2.7	2.8	2.7
Ta.9641.1.A1_a_at	Fructan 6-exohydrolase	2.8	1.9	2.1	3.2
TaAffx.122374.1.A1_at	CBFIVa-A2	2.2	1.7	1.4	2.7
TaAffx.128643.5.S1_at	TC428998 (POEI12)	4.4	1.5	4.1	1.9
TaAffx.129374.2.S1_at	Ice recrystallization inhibition protein 1 precursor	3.6	3.5	2.8	4.7
TaAffx.129374.2.S1_x_at	Ice recrystallization inhibition protein 1 precursor	4.3	4	3.5	5
TaAffx.130775.1.S1_at	Chromosome chr18 scaffold_1	2.5	2.2	1.9	1.5
TaAffx.131747.1.S1_x_at	Dehydrin	4.2	4.7	3.1	5.7
TaAffx.134872.1.S1_at	TC434386 (Ycf1)	1.1	1.3	1	1.1
TaAffx.144000.1.S1_s_at	Humulus lupulus 26S ribosomal RNA gene	5.6	2.3	5.8	1.9
TaAffx.17284.1.A1_at	Cold acclimation protein WCOR518	5.1	6.1	3.3	6.5
TaAffx.34169.1.S1_at	TC396129 (embryonic protein DC-8)	4.5	3.5	3.4	5
TaAffx.38397.1.A1_at	Expressed protein	3	3.3	3	3.1
TaAffx.38476.1.S1_at	Os03g0702000 protein	2.7	2	2.1	1.7
TaAffx.39904.1.A1_at	High light protein (jacalin like lectin)	2.7	2	1.5	2.8
TaAffx.54307.1.S1_x_at	Cold-responsive LEA/RAB-related COR protein	2.7	3.4	2.1	3.7
TaAffx.56641.1.A1_at	early nodulin 75-like protein	2.8	2.4	2.7	2.8
TaAffx.69918.1.S1_s_at	TC413630 (ATP/ADP-transporter)	1.7	1.7	1.8	1.5
TaAffx.70677.1.S1_at	Chaperone DnaJ-like protein	1.9	2	1.6	2
TaAffx.71465.1.S1_at	Serine/arginine repetitive matrix protein 1	3	2.8	2.3	3.5
TaAffx.73215.1.S1_at	TC407368	3.1	2.7	3.2	2.2
TaAffx.80153.1.S1_at	Ice recrystallization inhibition protein 1 precursor	3.1	3.4	2.9	3.5
TaAffx.92142.1.S1_at	TC397297 (glycosyltransferase)	3.4	2.7	2.7	1.6
TaAffx.95521.1.S1_at	Ice recrystallization inhibition protein 1 precursor	5.2	4.9	3.6	5.8
TaAffx.98930.1.A1_at	GB|EF028762.1|ABK55365.1 CBFIIId-12.1	3.2	2.8	2	2.6

Table S2: List of conserved and down-regulated genes in the four genotypes. This cluster of genes was obtained using the 3D time series gene expression data of the four wheat cultivars and the OPTricluster algorithm. Numbers in column 3-6 represent the fold change between the min and the max of the expression level across the experimental time points.

Probe ID	Description	wM	sN	sM	wN
Ta.10.2.S1_x_at	(1,3;1,4) beta glucanase precursor	-4.1	-3.9	-3.5	-3.1
Ta.14729.1.S1_at	TC382423	-5.8	-5.3	-5.7	-4.9
Ta.16407.1.S1_at	Cytochrome c	-4.6	-4.6	-4.2	-4.4
Ta.20509.1.S1_x_at	Expressed protein	-5.2	-4.4	-4.6	-4.4
Ta.20930.1.S1_at	Amylase inhibitor-like protein (DEF7)	-4.3	-4.5	-3.5	-4.1
Ta.21419.1.S1_at	Cold acclimation protein WCOR518	-1.1	-2.2	-2.1	-1.2
Ta.21419.1.S1_x_at	Cold acclimation protein WCOR518	-1	-2.1	-2	-1.2
Ta.23032.2.S1_a_at	TC437271 ()	-4.1	-3.9	-4.1	-3.7
Ta.23066.1.S1_s_at	Os07g0558900 protein	-5.6	-4.6	-5.4	-4.8
Ta.24155.2.S1_s_at	Early nodulin protein	-4.5	-2.7	-4.5	-2.8
Ta.24155.2.S1_x_at	Early nodulin protein	-4.1	-2.4	-4.2	-2.2
Ta.24544.1.S1_at	HS1-like protein	-4.7	-4.3	-4.8	-4
Ta.24630.2.S1_x_at	Anaeromyxobacter dehalogenans 2CP-C	-4.8	-4	-4.4	-3.3
Ta.28220.1.S1_at	DnaK-type molecular chaperone hsp70-rice	-4.5	-4.5	-5.4	-3.6
Ta.28271.1.S1_at	S-adenosylmethionine:tRNA ribosyltransferase	-5.2	-4	-5	-3.5
Ta.28394.3.S1_x_at	Auxin-repressed protein	-4.5	-3.6	-4.5	-2.8
Ta.28398.3.S1_a_at	Cold induced protein-like	-2.6	-2.6	-2.8	-2.2
Ta.28584.1.A1_at	FAD dependent oxidoreductase	-4	-3.1	-3.1	-4.1
Ta.28750.1.S1_at	Photosystem II 10 kDa polypeptide	-4.8	-4.4	-4.7	-4.5
Ta.28750.1.S1_x_at	Photosystem II 10 kDa polypeptide	-4.7	-4.2	-4.4	-4.3
Ta.28750.2.A1_x_at	Photosystem II 10 kDa polypeptide	-4.6	-4.4	-4.4	-4.8
Ta.28942.2.A1_at	1-aminocyclopropane-1-carboxylate oxidase	-4.1	-3.3	-4.3	-3.4
Ta.30827.1.A1_x_at	Jasmonate-induced protein (jacalin)	-0.7	-2.2	-2.4	0
Ta.350.1.A1_s_at	TaCBF6	-1.9	-2.8	-1.8	-2.6
Ta.4457.1.S1_at	SAM dependent carboxyl methyltransferase	-3.1	-3.5	-3.5	-1.7
Ta.4726.1.S1_at	Cyclopropane-fatty-acyl-phospholipid synthase	-2.1	-2	-2.6	-0.7
Ta.4830.2.A1_at	lipid-sensing domain containing protein	-1.5	-2.3	-2.3	-0.5
Ta.5198.2.S1_a_at	ABA-responsive protein-like	-3.9	-3.2	-4	-3.3
Ta.5208.1.S1_at	Thionin-like protein	-4.3	-1.3	-5.3	-2.5
Ta.5208.1.S1_x_at	Thionin-like protein	-4.3	-1.3	-5.2	-2.4
Ta.5539.1.S1_at	Extracellular invertase; glycosyl hydrolases	-5.1	-4.6	-5	-4.4
Ta.5539.3.A1_x_at	Extracellular invertase; glycosyl hydrolases	-5.6	-5.4	-5.6	-5.2
Ta.556.1.S1_at	Os05g0153300 protein	-4.7	-4.5	-2.5	-2.8
Ta.556.1.S1_x_at	Os05g0153300 protein	-4.4	-4.2	-2.5	-2.9
Ta.6098.1.S1_at	Hydroxyproline-rich glycoprotein-like	-4.1	-4.1	-4	-3.5
Ta.7143.2.S1_x_at	AGAP006957-PA, putative, expressed	-4.7	-4.8	-4.2	-5.5
Ta.7388.1.S1_at	Jasmonate-induced protein (jacalin-like)	-5.4	-1.1	-2.7	-4.1
Ta.7388.2.S1_a_at	Jasmonate-induced protein (jacalin-like)	-5.8	-1.8	-2.7	-5.5
Ta.7388.2.S1_x_at	Jasmonate-induced protein (jacalin-like)	-5.2	-1.3	-2.7	-4.7
Ta.7406.1.S1_x_at	Haloacid dehalogenase-like hydrolase (HAD)	-4.4	-3.8	-3.8	-4
Ta.7406.2.S1_at	Haloacid dehalogenase-like hydrolase (HAD)	-4.4	-3.8	-3.9	-4.1
Ta.7406.2.S1_x_at	Haloacid dehalogenase-like hydrolase (HAD)	-4.3	-3.6	-3.8	-4.1
Ta.7883.1.S1_x_at	dirigent, putative, expressed	-4.5	-5.7	-3.2	-4
Ta.7915.1.A1_at	Zinc finger protein-like	-4.2	-5.1	-4.3	-2.3
Ta.7963.2.S1_x_at	dirigent, putative	-4.9	-6	-5	-4
Ta.8082.1.A1_x_at	Cyclopropane-fatty-acyl-phospholipid synthase	-2	-2.1	-2.9	-0.4
Ta.9000.1.S1_at	Acid beta-fructofuranosidase precursor	-5.2	-4.8	-5.3	-4.9
Ta.9513.1.S1_a_at	Cold induced protein-like	-2.3	-2.1	-3.3	-1.7
Ta.9538.1.A1_at	fatty acid amide hydrolase	-1.5	-2.3	-2.3	-0.8
Ta.9664.1.A1_at	Cyclopropane-fatty-acyl-phospholipid synthase	-4.2	-5.9	-4.5	-3.3
Ta.9711.1.S1_at	23 kDa jasmonate-induced protein	-4.4	-4.1	-4.3	-3.3
Ta.9717.1.A1_a_at	Flavonoid 7-O-methyltransferase-like	-8	-3.5	-6.9	-3.3
Ta.9717.1.A1_x_at	Flavonoid 7-O-methyltransferase-like	-6.6	-1.7	-5.8	-2
TaAffx.106168.1.S1_x_at	ABA-responsive protein-like	-2.3	-2	-2.4	-1.8
TaAffx.12929.1.A1_at	3-ketoacyl-CoA synthase 17	-1.2	-2.4	-1.9	-0.9
TaAffx.15961.1.S1_at	CBFIIIc-B10	-1.9	-3.3	-1.4	-2.8
TaAffx.24365.1.S1_at	Cyclopropane-fatty-acyl-phospholipid synthase	-1.9	-2.6	-3.6	-0.6
TaAffx.28156.1.S1_at	CA664383	-4.7	-4.6	-4.6	-4.1
TaAffx.43393.1.S1_at	RuBisCo subunit binding-protein beta subunit	-6.8	-5.6	-6.2	-5.5
TaAffx.43752.1.A1_at	CBFIIIc-D3	-3.3	-4.2	-3.5	-4

TaAffx.616.2.S1_s_at	Cold induced protein-like	-2.9	-2.4	-3.1	-2.1
TaAffx.92408.1.S1_at	Cyclopropane fatty acid synthase	-2.4	-3.2	-3.1	-1.3
TaAffx.98394.1.S1_at	Zinc finger, C2H2 type family protein (ZAT10)	-1.9	-2.5	-2	-2.1

Table S3: List of selected significant divergent genes and their max fold change. This cluster of genes was obtained using the 3D time series gene expression data of the four wheat cultivars and the OPTricluster algorithm. Numbers in column 3-6 represent the fold change between the min and the max of the expression level across the experimental time points.

Probe ID	Description	wM*	sN*	sM*	wN*
AFFX-Ta_18SrRNA_at	18S rRNA gene for 18S ribosomal RNA /LEN=1764	3.3	1.3	3	1.1
Ta.10782.1.S1_at	Cluster: FT-like protein	0.3	2.7	2.5	0.3
Ta.11076.1.A1_x_at	TC442001	0.4	1.7	0	2.3
Ta.11152.2.A1_a_at	Cluster: Sucrose synthase 3	1.2	2.2	0.9	3.2
Ta.11152.3.S1_at	Cluster: Sucrose synthase 3	1.4	2.1	0.8	3.1
Ta.11539.1.A1_at	TC438076	2.6	1.9	2.5	0.4
Ta.123.1.S1_x_at	Cluster: Cold acclimation protein WCOR80	5.2	5.8	4.3	6.7
Ta.12413.1.S1_at	Cluster: Acid phosphatase	0.1	1.2	0.1	2.7
Ta.12657.1.S1_at	Cluster: LEA protein 12	0.6	2.2	0.2	2.9
Ta.13008.1.S1_at	Cluster: Galactinol synthase	2.7	2.6	1.5	4
Ta.13153.1.S1_s_at	Cluster: Biotin synthesis protein	0.2	2.2	0.2	1.4
Ta.13165.3.S1_x_at	late-embryogenesis abundant protein	1.6	2.5	0.7	3.1
Ta.13183.1.S1_s_at	Cluster: Cold regulated protein	3.4	4.1	2.5	5.1
Ta.13183.1.S1_x_at	Cluster: Cold regulated protein	4.2	4.7	3.1	6.5
Ta.13232.1.S1_at	Cluster: Sterol desaturase family protein	2.4	3.4	1.4	3.4
Ta.13232.2.S1_at	Cluster: Sterol desaturase family protein	2	2.1	0.7	4
Ta.13255.1.S1_at	Cluster: Dehydrin 7	2.2	2.9	0.3	5.5
Ta.13318.1.S1_at	Cluster: GABA-A receptor epsilon-like subunit	2.3	1.6	3.1	0.6
Ta.13334.1.A1_at	Hessian fly response gene 1 protein (Jacalin-like)	0	2.2	0	0
Ta.13337.1.S1_at	Cluster: Xyloglucan endotransglycosylase	0.1	0.1	0	2
Ta.13396.1.S1_at	late embryogenesis abundant group 1	0.7	2.6	0.4	3.7
Ta.13715.1.S1_s_at	Cluster: HFR-3; n=1	0	3.1	0	0.7
Ta.13784.1.S1_at	Cluster: BLT14.1 protein	5.4	5.7	4	6.4
Ta.13808.1.S1_at	TC405851	0.1	2.2	0.3	1.2
Ta.14071.1.S1_x_at	integral membrane protein DUF6 containing protein	0.1	0.1	0	2.5
Ta.14145.1.S1_at	Cluster: Seed maturation protein	2.1	3.3	1.5	3.6
Ta.142.1.S1_at	Cluster: MADS-box protein TaVRT-1 (VNR-B1)	3.3	5.1	4.7	2.9
Ta.14568.1.S1_at	Cluster: Amylase inhibitor-like protein	0	2	0.6	0
Ta.14903.1.S1_at	Cluster: Chalcone synthase	4.6	2.3	4.1	1.7
Ta.14946.1.S1_at	Cluster: Chitinase 1	2	0.3	3.7	0.3
Ta.14995.2.S1_a_at	Cluster: Xyloglucan endotransglucosylase/hydrolase	0.2	1.2	0	2.1
Ta.14995.2.S1_x_at	Cluster: Xyloglucan endotransglucosylase/hydrolase	0	1.1	0.1	2.1
Ta.15313.2.A1_a_at	Cluster: Predicted protein	0.7	0.7	2.6	0
Ta.16845.1.S1_at	TC426385	0.3	2.3	0.1	2.4
Ta.16907.1.S1_at	Cluster: WRKY45 transcription factor	0	2.6	0.2	0.4
Ta.18186.1.A1_at	LTPL114 - Protease inhibitor/seed storage/LTP	1.4	3.1	1.2	3.4
Ta.1840.1.S1_a_at	Cluster: Protease inhibitor-like protein	0	2	0	1.4
Ta.1840.1.S1_at	Cluster: Protease inhibitor-like protein	0	2.3	0.2	1.6
Ta.1840.1.S1_x_at	Cluster: Protease inhibitor-like protein	0	2.1	0	1.4
Ta.19206.1.A1_at	Putative lipid tranfer protein	0.6	2.6	0.9	1.2
Ta.19303.1.S1_at	Cluster: Expressed protein	3.1	4.3	2.8	4.9
Ta.20102.2.S1_at	Cluster: Polyphenol oxidase	2.8	0.7	2.4	0.6
Ta.20195.1.S1_x_at	Cluster: Glycine rich protein	0.7	0.3	0.1	2.8
Ta.20519.1.S1_at	LTPL114 - Protease inhibitor/seed storage/LTP	0.8	4.1	0.6	5.6
Ta.20570.1.A1_at	Cluster: 1-aminocyclopropane-1-carboxylate oxidase	0.2	2.1	1	0
Ta.20756.1.S1_at	Cluster: Physical impedance induced protein	0.3	3.6	1.1	1.5
Ta.20756.1.S1_x_at	Cluster: Physical impedance induced protein	0.1	3.6	1	1.8
Ta.21297.1.S1_at	Cluster: Beta-glucanase; n=1	1.3	1.5	0.9	2.9
Ta.22050.1.S1_at	Cluster: RNA polymerase sigma factor	0.7	0.7	0	2.9
Ta.22628.1.S1_at	TC436963	1	2.2	0.7	3.2
Ta.22628.1.S1_x_at	TC436963	1	2.1	0.7	2.8
Ta.22968.1.S1_at	Lipid transfer protein-like protein	1.9	3.6	1.3	3.6
Ta.23230.1.S1_at	TC438316	1.9	1.7	1.1	3.4
Ta.23322.3.S1_at	Cluster: Thaumatin-like protein TLP8	0.3	3	0.1	2.6
Ta.23327.1.S1_at	Cluster: UBA/THIF-type NAD/FAD binding fold	2.1	1.3	1.4	3.8
Ta.23797.1.S1_x_at	Cluster: LEA1 protein	1.6	2.8	0.8	2.8
Ta.24158.1.S1_a_at	embryonic protein DC-8, putative, expressed,	2.2	2.8	0.9	4.4
Ta.24591.1.A1_at	Cluster: Chromosome undetermined scaffold_314	0.5	0.8	2.1	0
Ta.25089.1.S1_s_at	Cluster: LEA protein 12	0.6	1.9	0.3	2.4
Ta.25678.1.A1_at	Cluster: Beta-glucosidase aggregating factor 1	1.7	3.4	3.3	0.6

Ta.26049.1.S1_a_at	Cluster: Transcription factor Myb1	0	1.1	0	2.3
Ta.2638.1.S1_at	Cluster: Rab protein; n=1	0	1.1	0	3.1
Ta.2704.1.S1_at	Cluster: Dehydrin Rab15	1.4	2.1	0.6	2.9
Ta.2704.1.S1_x_at	Cluster: Dehydrin Rab15	1.4	2.1	0.6	2.8
Ta.27389.2.S1_x_at	Cluster: Gamma-2-purothionin	5.3	4.5	5.4	3.4
Ta.2787.1.S1_at	Cluster: Dehydrin WZY1-1	1.4	2.1	0.8	3.6
Ta.28437.2.S1_s_at	Cluster: Proline-rich protein precursor (POEI12)	2.2	0	1.3	0
Ta.28533.1.S1_at	Cluster: PS II 10 kDa protein	1.9	2.7	1.8	4.7
Ta.28605.1.S1_at	Cluster: LEA2 protein	2.4	4.2	1.6	3.7
Ta.28613.1.S1_at	Cluster: Cold acclimation protein WCOR726	2.9	5.7	2.2	6.8
Ta.28827.1.S1_at	Cluster: Histone H1	1.7	2.7	1.1	3.3
Ta.28848.1.S1_at	Cluster: LEA protein 12	0.4	1.5	0.4	2.6
Ta.28983.2.S1_at	Cluster: Glycine-rich cell wall structural protein	0.1	0.9	0.3	3
Ta.28983.2.S1_x_at	Cluster: Glycine-rich cell wall structural protein	0.1	0.9	0.1	3.2
Ta.30113.1.S1_at	Cluster: Galactinol synthase	2.9	2.8	1.4	4.5
Ta.30509.1.A1_at	Cluster: Glycosyltransferase	2.4	3	1.1	4.7
Ta.30607.1.A1_at	Cluster: VRN-A1	5	0.9	0.7	4.3
Ta.30827.1.A1_x_at	Cluster: Jasmonate-induced protein (jacalin-like)	0.1	2.2	0	2.4
Ta.351.2.S1_x_at	Cluster: Cold acclimation induced protein 2-1	1.6	2.3	1.2	3.8
Ta.3583.1.A1_at	Cluster: MADS-box transcription factor TaAGL29	0.1	3	2.9	0.2
Ta.4035.3.S1_at	Os9bglu31 - beta-glucosidase, dhurrinase	2.4	2.5	1.6	4.1
Ta.425.1.S1_s_at	Cluster: 1-aminocyclopropane-1-carboxylate oxidase	0.1	2.3	1.5	0.3
Ta.5148.1.S1_a_at	xylem cysteine proteinase 2 precursor	2.1	0.8	2.1	0
Ta.5186.1.S1_at	TC411740	0.6	3.3	0.5	3
Ta.5888.1.S1_s_at	Cluster: Polar amino acid ABC transporter (LEA)	3.1	3.8	1.3	5.5
Ta.6019.1.S1_at	Anaeromyxobacter dehalogenans (LEA)	1.9	2.4	1	3.6
Ta.6146.1.S1_at	similar to late embryogenesis abundant proteins	0.4	1.4	0.1	3.1
Ta.6174.1.S1_at	Hydroxyproline-rich glycoprotein DZ-HRGP	1.2	1.8	1	3.3
Ta.6374.3.S1_a_at	18S small subunit ribosomal RNA gene	0.8	1.7	2.5	0
Ta.6793.1.A1_at	Cluster: MADS2	0.8	3.7	3.6	0.5
Ta.7223.2.S1_at	flavonol synthase/flavanone 3-hydroxylase	0	2.1	0	0.6
Ta.7223.3.S1_x_at	flavonol synthase/flavanone 3-hydroxylase	0	2.4	0	1
Ta.7330.1.S1_a_at	TC398487	0.3	0.9	2.1	0
Ta.758.1.A1_at	Cluster: HFR-3	1.8	2.6	3.5	0.6
Ta.8037.1.A1_at	Ice recrystallization inhibition protein 1 precursor	5.5	5.5	4.2	6.3
Ta.8262.1.S1_at	cytochrome P450, putative, expressed	3.1	0.7	2.3	1.3
Ta.8614.1.S1_at	Cluster: WRKY45 transcription factor	0	2	0.1	0.1
Ta.87.1.S1_x_at	Cluster: PSBGer1 protein	0.4	2	0	2.1
TaAffx.104648.1.S1_at	Cluster: Pathogenesis-related protein precursor	2.3	1.8	1.4	3.8
TaAffx.108353.1.S1_at	Cluster: Chalcone synthase	3.6	0.6	3.2	1
TaAffx.110973.1.S1_at	Cluster: Seed maturation protein	1.6	2.5	0.5	2.6
TaAffx.116865.2.S1_at	LTPL114 - Protease inhibitor/seed storage/LTP	4.8	5.3	3.7	6
TaAffx.120063.1.S1_at	Cluster: MADS2	0.9	4.6	4.2	0.3
TaAffx.120063.2.S1_s_at	Cluster: MADS2	0.9	4.2	3.9	0.3
TaAffx.124475.1.A1_at	Cluster: Hblt14.2 protein	2.9	4.1	1.4	6.4
TaAffx.128555.1.S1_at	Cluster: Dehydrin DHN3	0.5	2.7	0	4.3
TaAffx.128643.3.S1_at	Cluster: Proline-rich protein precursor (POEI13)	2.8	0	1.4	0.4
TaAffx.128643.5.S1_at	POEI12 - Pollen Ole e I allergen and extensin	4.4	1.5	3.4	1.9
TaAffx.131747.1.S1_x_at	Cluster: Dehydrin	4.2	4.7	3.1	5.7
TaAffx.132335.1.A1_at	CK213289	3.1	3.8	1.8	4.5
TaAffx.137429.1.S1_at	Cluster: COR39	0.5	1.5	0.4	2.5
TaAffx.143995.17.S1_s_at	Cluster: VRN-A1	5.2	1.7	1.5	5.1
TaAffx.144000.1.S1_s_at	Humulus lupulus 26S	5.6	2.3	5.8	1.9
TaAffx.144000.1.S1_x_at	Humulus lupulus 26S	5.2	2	5.1	1.4
TaAffx.15327.1.S1_at	Cluster: Beta-1,3-glucanase	3.6	4.6	4.5	5.6
TaAffx.17284.1.A1_at	Cluster: Cold acclimation protein WCOR518	5.1	6.1	3.3	6.4
TaAffx.34169.1.S1_at	TC396129	4.5	3.5	2.8	5
TaAffx.3462.1.S1_at	Cluster: Cold acclimation induced protein 2-1	1.8	2.3	1.4	3.9
TaAffx.37494.1.A1_at	Cluster: Os03g0430500 protein	0.8	1.7	2.2	0
TaAffx.38460.1.S1_at	jacalin-like lectin domain containing protein	1.4	3.5	1.5	1.7
TaAffx.46097.2.S1_at	Cluster: Dehydrin 1	1.2	1.4	0.3	2.4
TaAffx.59867.1.S1_at	Cluster: 1-aminocyclopropane-1-carboxylate oxidase	0.3	2.8	0.9	0.5
TaAffx.70609.1.S1_at	Cluster: Caffeic acid O-methyltransferase	1.7	0	2.1	0.1
TaAffx.8335.1.S1_at	Cluster: Receptor-like kinase	2.6	2.2	1.3	3.6
TaAffx.92706.1.A1_at	Cluster: MADS2	0.8	3.6	3.3	0.2
TaAffx.93127.1.A1_at	Cluster: Shhikimate 5-dehydrogenase	0.1	2.1	0.2	1.6
TaAffx.95521.1.S1_at	Ice recrystallization inhibition protein 1 precursor	5.2	4.9	3.6	5.8

*Non-shaded cells represent cultivars under which the corresponding probe is upregulated. Thus may correspond to potential markers.

Table S4: List of selected genes and their max fold change present in this study and not in [3]. This cluster of genes was obtained by comparing the set of genes identified in this study with the set of genes identified in [3]. Numbers in column 3-6 represent the fold change between the min and the max of the expression level across the experimental time points.

AffyID	Symbols/definitions	wM	sN	sM	wN
Ta.123.1.S1_x_at	Cold acclimation protein WCOR80	5.5	6.0	4.3	6.7
Ta.124.1.S1_x_at	Cold-shock protein CS120	5.1	4.6	4.0	4.7
Ta.13070.1.S1_at	Non-specific lipid transfer protein 1 precursor	2.5	2.5	1.9	2.6
Ta.13070.2.S1_a_at	Non-specific lipid transfer protein 1 precursor	2.3	2.5	1.5	2.9
Ta.13070.2.S1_x_at	Non-specific lipid transfer protein 1 precursor	1.6	2.0	0.9	2.2
Ta.13183.1.S1_s_at	Cold regulated protein	3.3	4.1	2.7	5.0
Ta.13183.1.S1_x_at	Cold regulated protein	4.2	4.7	3.3	6.4
Ta.14492.1.S1_at	Lipid transfer protein-like protein	1.7	1.5	2.0	1.0
Ta.145.1.A1_x_at	Cold shock protein CS66	4.5	5.0	3.8	5.5
Ta.1526.1.S1_at	Type 1 non specific lipid transfer protein precursor	1.4	1.4	1.3	2.1
Ta.1526.1.S1_x_at	Type 1 non specific lipid transfer protein precursor	1.3	1.4	1.3	2.1
Ta.1840.1.S1_a_at	Protease inhibitor-like protein	2.3	3.3	1.8	2.8
Ta.1840.1.S1_at	Protease inhibitor-like protein	2.0	3.2	1.8	2.9
Ta.1840.1.S1_x_at	Protease inhibitor-like protein	1.9	3.4	1.8	2.8
Ta.18487.1.S1_x_at	Cold-responsive protein	2.3	1.8	1.9	2.6
Ta.18647.1.S1_s_at	Nonspecific lipid transfer protein	1.3	2.1	1.8	2.0
Ta.19206.1.A1_at	Putative lipid tranfer protein [	1.3	2.6	1.5	2.1
Ta.21419.1.S1_at	Cold acclimation protein WCOR518	2.3	2.8	2.9	2.0
Ta.21419.1.S1_x_at	Cold acclimation protein WCOR518	2.2	2.9	2.7	2.1
Ta.21419.2.S1_at	Cold acclimation protein WCOR518	2.0	1.3	1.4	1.5
Ta.2148.1.S1_x_at	Cold-responsive protein	2.3	2.4	2.1	3.1
Ta.21508.1.A1_a_at	Cold induced protein-like	2.7	2.1	2.6	2.5
Ta.21508.2.S1_x_at	Cold induced protein-like	2.1	1.8	2.0	1.9
Ta.21766.1.S1_at	PTACR7	4.0	4.0	4.3	3.9
Ta.22333.1.S1_at	Fatty acyl coA reductase	1.9	2.5	2.2	1.3
Ta.22968.1.S1_at	Lipid transfer protein-like protein	1.9	3.6	1.8	3.6
Ta.23758.1.S1_x_at	Cold acclimation protein WCOR518	3.2	2.6	2.9	2.7
Ta.2541.1.S1_s_at	Cold acclimation protein WCOR615	2.8	2.5	2.6	2.4
Ta.2541.1.S1_x_at	Cold acclimation protein WCOR615	3.6	3.6	3.1	3.8
Ta.255.1.S1_at	Lipid transfer protein precursor	1.6	1.7	1.5	2.5
Ta.26144.1.A1_at	FAR1 | fatty acid reductase 1	2.5	1.6	1.7	2.4
Ta.26144.1.A1_s_at	FAR1 | fatty acid reductase 1	2.9	1.6	2.3	2.4
Ta.27168.1.S1_at	Lipid transfer protein-like protein	2.2	2.4	1.3	2.9
Ta.28327.1.S1_x_at	Type 2 non specific lipid transfer protein precursor	1.9	1.7	2.0	2.6
Ta.28398.3.S1_a_at	Cold induced protein-like	2.6	2.5	2.8	2.2
Ta.28917.1.S1_x_at	Cold acclimation protein WCOR518	3.2	2.5	2.7	2.6
Ta.30336.1.S1_x_at	Cold-shock protein CS120	6.2	6.1	4.8	5.9
Ta.351.2.S1_x_at	Cold acclimation induced protein 2-1	2.0	3.4	2.1	4.1
Ta.5348.1.S1_x_at	Lipid transfer protein-like protein	1.4	3.1	0.6	1.1
Ta.7479.2.S1_x_at	Cold-regulated protein BLT14	3.1	3.1	3.1	2.9
Ta.7479.3.S1_x_at	Cold-regulated protein BLT14	2.5	2.7	2.8	2.5
Ta.8082.1.A1_x_at	fatty-acyl-phospholipid synthase, putative, expressed	2.1	2.0	2.8	0.3
Ta.9767.2.S1_at	Putative very-long-chain fatty acid condensing enzyme CUT1	1.6	2.1	1.9	1.4
Ta.994.1.S1_at	Lipid transfer protein-like protein	1.4	2.1	2.4	1.8
Ta.994.1.S1_x_at	Lipid transfer protein-like protein	1.4	2.1	2.3	1.8
